# Close ecological relationship among species facilitated horizontal transfer of retrotransposons

**DOI:** 10.1186/s12862-016-0767-0

**Published:** 2016-10-07

**Authors:** Xianzong Wang, Xiaolin Liu

**Affiliations:** Shaanxi Key Laboratory of Molecular Biology for Agriculture, College of Animal Science and Technology, Northwest A&F University, Yangling, 712100 Shaanxi People’s Republic of China

**Keywords:** Horizontal transfer, Retrotransposon, Crustacean, Parasites, Ecological relationship, Predation

## Abstract

**Background:**

Horizontal transfer (HT) of genetic materials is increasingly being found in both animals and plants and mainly concerns transposable elements (TEs). Many crustaceans have big genome sizes and are thus likely to harbor high TE contents. Their habitat might offer them ample opportunities to exchange genetic materials with organisms that are ecologically close but taxonomically distant to them.

**Results:**

In this study, we analyzed the transcriptome of Pacific white shrimp (*Litopenaeus vannamei*), an important economic crustacean, to explore traces of HT events. From a collection of newly assembled transcripts, we identified 395 high reliable TE transcripts, most of which were retrotransposon transcripts. One hundred fifty-seven of those transcripts showed highest similarity to sequences from non-arthropod organisms, including ray-finned fishes, mollusks and putative parasites. In total, 16 already known *L. vannamei* TE families are likely to be involved in horizontal transfer events. Phylogenetic analyses of 10 *L. vannamei* TE families and their homologues (protein sequences) revealed that *L. vannamei* TE families were generally more close to sequences from aquatic species. Furthermore, TEs from other aquatic species also tend to group together, although they are often distantly related in taxonomy. Sequences from parasites and microorganisms were also widely present, indicating their possible important roles in HT events. Expression profile analyses of transcripts in two NCBI BioProjects revealed that transcripts involved in HT events are likely to play important roles in antiviral immunity. More specifically, those transcripts might act as inhibitors of antiviral immunity.

**Conclusions:**

Close ecological relationship, especially predation, might greatly facilitate HT events among aquatic species. This could be achieved through exchange of parasites and microorganisms, or through direct DNA flow. The occurrence of HT events may be largely incidental, but the effects could be beneficial for recipients.

**Electronic supplementary material:**

The online version of this article (doi:10.1186/s12862-016-0767-0) contains supplementary material, which is available to authorized users.

## Background

Horizontal transfer (HT) of genetic materials between reproductively isolated species is an important mechanism in the evolution of prokaryotic genomes [1–3]. Recent studies showed that HT events are also widespread in animals and plants and mainly concern transposable elements (TEs) [4–12]. TEs are usually grouped into two distinct classes: class I elements (retrotransposons) and class II elements (DNA transposons) [13]. Retrotransposons, which integrate into new sites via a copy and paste mechanism, are often the major components in the genomes of many eukaryotic species, especially those with large genomes [14]. Retrotransposons constitute over 50 % of the genomes in many plants [15]. In mammals, LINE-1 (L1) retrotransposons’ activity alone generated at least 20 % of the genome [16]. The horizontally transferred TEs are also mainly retrotransposons [6, 11]. However, unlike retroviruses, retrotransposons do not encode an envelope protein and hence require a vector between species to transpose horizontally. The vector discussed here is often thought to be parasites, which have ample opportunities to exchange genetic material with their hosts as the result of an intimate, long-term physical association [12]. In eukaryotes, the underlying mechanisms are largely unknown, but the proximity of species is almost indispensable in all HT events and may consequently increase the likelihood of HT. If HT also plays an important role in eukaryotic evolution, we may expect to find more evidence of HT events among species that are distantly related in taxonomy yet live in the same habitat.

The ancient crustaceans are a great model to investigate horizontal TE transfer (HTT) in eukaryotes. Many of them have big genome sizes and are thus likely to harbor high TE contents [17]. Decapod crustaceans, for instance, have genome sizes range from 1.05 Gb to 40 Gb (for human, the value is around 3 Gb). They have ample opportunities to intimately connect with fishes, mollusks and other animas that also inhabit in fresh or salty water. Furthermore, this connection is much less disturbed by geographical isolation when compared to land animals. Therefore, crustaceans may at least have some sequences that show higher similarity to other aquatic animals than land arthropods. However, one big drawback is that the whole genome sequencing projects of most crustaceans are not finished yet. Even though, next generation sequencing has made available more comprehensive transcriptome sequences for many crustaceans [18–20]. And HTTs detected in transcriptome are of particular importance: they are still active and may still have impact on genome evolution.

In this study, we particularly focused on Pacific white shrimp, *Litopenaeus vannamei*. This species has a genome size approximately 70 % of the human genome and is likely to harbor high TE content [21]. Due to its high commercial value, extensive efforts have been made on its transcriptomics to better understand its immunity, growth and development [18, 22]. We identified hundreds of reliable TE fragments from an up-to-date transcriptome assembly of *L. vannamei* and showed that many of them are involved in HTT events.

## Results and discussion

### Overview of TE transcripts in *L. vannamei* transcriptome

We identified 395 TE transcripts in total, all of which have transposon-related conserved domains and their actual existence could be confirmed by sequence similarity search against whole collection of *L. vannamei* ESTs and nucleotides (mostly mRNA/cDNA). Furthermore, we ensured that they are not transcripts of single/low copy genes that happened to contain TE-related domains, e.g., the *L. vannamei* elongation factor 2 (EF2, GenBank ID: GU136230.1) mRNA contains a conserved domain that is a member of the TetM_like subfamily (NCBI CDD accession number: cd04168), which are typically found on mobile genetic elements. Of the 395 transcripts, 380 could be identified as transcripts of retrotransposons, 284 of which were further identified as Non-LTR retrotransposon transcripts (Table 1 and Additional file 1). The corresponding superfamilies of Non-LTR retrotransposon transcripts were also more diverse than LTR retrotransposon transcripts. Two hundred thirty transcripts could be identified as transcripts of already known *L. vannamei* TE families. It should be noted that two families, Gypsy-3_LVa-LTR and Penelope-6_LVa, were not consistent with their identified superfamilies. This is possibly the results of nested TEs (the insertion of TEs into pre-existing TEs), especially for the corresponding transcript of Gypsy-3_LVa-LTR, which contains a conserved RT-nLTR domain and consequently resulted in the identification of superfamily as RTE.

**Table 1:** Classification of 395 TE transcripts in *L. vannamei* transcriptome

Group	Superfamily/clade	Number	Sum	Family/consensus sequence (number of transcripts)
DNA transposon	EnSpm/CACTA	1		-
	Harbinger	2		Harbinger-N1_LVa (2)
	Mariner/Tc1	1		-
	hAT	1		-
	Unknown	4	9	DNA8-1_LVa (2)
LTR retrotransposon	BEL	33		BEL-1_LVa-I (6), BEL-2_LVa (1)
	Copia	6		-
	DIRS	1		-
	Gypsy	56	96	Gypsy-12_LVa-I (3), Gypsy-14_LVa-I (3), Gypsy-16_LVa (3), Gypsy-17_LVa (4), Gypsy-18_LVa (1), Gypsy-1_LVa-I (1), Gypsy-3_LVa-I (1), Gypsy-4_LVa-I (7), Gypsy-5_LVa-I (1)
Non-LTR retrotransposon	CR1	50		Penelope-6_LVa (4)
	Crack	2		-
	Daphne	3		-
	I	3		-
	Ingi	15		Ingi-1_LVa (5)
	Jockey	1		Jockey-1_LVa (1)
	Kiri	1		-
	L2B	1		-
	Nimb	53		Nimb-N2_LVa (1), Nimb-2_LVa (6), Nimb-1_LVa (30)
	Penelope	52		Penelope-1_LVa (31), Penelope-2_LVa (5), Penelope-3_LVa (8), Penelope-4_LVa (3), Penelope-5_LVa (2), Penelope-8_LVa (1)
	RTE	93		RTE-1_LVa (2), RTE-2_LVa (20), RTE-3_LVa (66), Gypsy-3_LVa-LTR (1)
	RTEX	1		-
	Unknown	9	284	NonLTR-1_LVa (8)
Unknown	Unknown	6	6	TE-1_LVa (1)

### *L. vannamei* TE transcripts showed high similarity to nucleotide sequences from distantly related aquatic species

By querying against NCBI BLAST Nucleotide database, we found that 244 transcripts had significant hits (*E*-value < 1e-5). The taxa of organisms present in top hits were extracted and counted. In total, 17 taxa were used to distinguish different species and evaluate their relationships. As shown is Table 2, arthropods were the most frequent top hits, followed by ray-finned fishes (actinopterygii) and mollusks. Species in cnidaria, nematoda and platyhelminthes, many of which are well known parasites, were also present in top hits with considerable number. Overall speaking, species from top hits represented a wide range of taxa, but most of them either also live in salty/fresh water or are potential parasites. Exceptions come from plants, mammals and birds; however, their frequencies as top hits are very low. It is noteworthy that as many as 30 transcripts showed high similarity to sequences from viruses. Further analysis revealed that they are actually all transcripts of Penelope-1_LVa, which contains fragments of white spot syndrome virus (WSSV). WSSV is one of the most fatal threats to shrimp farming throughout the globe [23, 24]; therefore, future studies on this TE family might afford novel perspective for antiviral research.

**Table 2:** Taxa of TE transcripts’ top hits in querying against NCBI BLAST Nucleotide database

Group	Superfamily	Arthropoda	Actinopterygii	Mollusca	Echinodermata	Brachiopoda	Enteropneusta	Annelida	Alveolata	Cyclostomata	Cnidaria	Nematoda	Platyhelminthes	Bacteria	Viruses	Embryophyta	Mammalia	Aves	Total (number of transcripts)
Non-LTR retrotransposon	Ingi								1										1
	Nimb	6	2										1	1			2	1	13
	Crack		1																1
	RTE	63	1	5	1	3	1			3		6					1		84
	Daphne		1										1						2
	CR1	11	7								8						1		27
	Penelope		1								1		1		30				33
LTR retrotransposon	BEL		3	23		6													32
	Copia		5																5
	Gypsy	5	20				4						7			3			39
DNA transposon	hAT	1																	1
	EnSpm							1											1
Unknown	Unknown	1	2									2							5
Total (number of transcripts)		87	43	28	1	9	5	1	1	3	9	8	10	1	30	3	4	1	244

Most transcripts that match to arthropods in top place were transcripts of Non-LTR retrotransposons, especially the RTE superfamilies, while those match to ray-finned fishes and mollusks in top place were mainly transcripts of LTR retrotransposons. The overall transcripts of *L. vannamei*, however, are mainly arthropod conservative [18]. A simplest explanation for this phylogenetic incongruence is that transcripts which matched non-arthropod species in top place are involved in HTTs. Of 157 such transcripts, 83 could be identified as transcripts of already known *L. vannamei* TE families. There are 16 such TE families in total, which were used to query the NCBI BLAST chromosome and HTGS databases in order to find presence of their homologues in genomes of other species. As shown in Table 3, for query sequences that have significant hits, their top hits were also mostly from aquatic species. Yet it should be noted that query coverage was very low for every query sequence, making it impossible to get nucleotide homologues long enough for phylogenetic analyses. Furthermore, nearly half of the 16 TE families did not have significant hits. These suggest that the common ancestors of the 16 TE families and their homologues have diverged greatly among species. Consequently, the top hits of TE families may not be their nearest neighbor in phylogenies [25] and stronger evidences of HTT are needed.

**Table 3:** Top hits of 16 *L. vannamei* TE families in querying against chromosome and HTGS databases

	Taxon	Organism	*E*-value	Identity (%)	Query coverage (%)
BEL-1_LVa-I	Brachiopoda	*Lingula anatina*	8.63e-30	70	6
Gypsy-14_LVa-I	Mollusca	*Lottia gigantea*	9.36e-29	70	6
Gypsy-17_LVa	Actinopterygii	*Danio rerio*	3.21e-11	84	3
Gypsy-3_LVa-LTR	Actinopterygii	*Salmo salar*	1.00e-43	70	30
Gypsy-4_LVa-I	Nematoda	*Trichinella spiralis*	1.90e-23	72	7
Penelope-6_LVa	Arthropoda	*Limulus polyphemus*	7.99e-14	73	6
RTE-1_LVa	Actinopterygii	*Oryzias latipes*	1.86e-28	66	22
RTE-2_LVa	Echinodermata	*Strongylocentrotus purpuratus*	1.31e-45	65	21
RTE-3_LVa	Echinodermata	*Strongylocentrotus purpuratus*	6.34e-48	80	7
Gypsy-18_LVa, Gypsy-5_LVa-I , Nimb-1_LVa, Nimb-2_LVa, Penelope-1_LVa, Penelope-3_LVa, Penelope-8_LVa have no significant hit (*E*-value < 1e-10)					

### Phylogenetic incongruence of TEs are closely linked with ecological relationships among species

To tackle the above problem, we used the protein sequences of 10 *L. vannamei* TE families (Table 4 and Additional file 2; the remaining six families do not have annotated protein sequences) to query the NCBI BLAST protein database, in order to find hits with higher query coverage (>60 %). Phylogenetic analysis using maximum likelihood method was conducted for each query sequence and its significant hits (E-value at 0). We used FastTree and RAxML (RAxML trees are provided in Additional file 3; Additional files 4, 5, 6, 7, 8, 9, 10 are FastTree trees) to infer phylogenetic trees [26, 27]. Both methods gave similar topologies around *L. vannamei* sequences. Only Nimb-2_LVa and RTE-3_LVa have different closest neighbors between the two methods. Of the 10 *L. vannamei* TE families, seven were most closely related to non-arthropod aquatic animals and only three were most closely related to insects (Nimb-1_LVa, Nimb-2_LVa and RTE-1_LVa). In addition to further confirming that many *L. vannamei* TE families are involved in HTT events, there are also more interesting details.

**Table 4:** Protein sequences of 10 *L. vannamei* TE families used for blastp search

	Length (aa)	Conserved domain	Accession	Interval	*E*-value
BEL-1_LVa-I	1413	RT_pepA17	cd01644	341–549	1.03e-68
		Peptidase_A17 super family	cl05112	567–771	4.02e-50
		pepsin_retropepsin_like super family	cl11403	49–203	3.20e-14
		rve	pfam00665	1079–1179	1.24e-06
Gypsy-14_LVa-I	874	rve	pfam00665	765–870	4.32e-16
Nimb-1_LVa	1286	RT_nLTR_like	cd01650	520–777	1.19e-44
		Rnase_HI_RT_non_LTR	cd09276	991–1112	5.91e-26
		EEP super family	cl00490	97–240	5.03e-27
		RVT_1	pfam00078	528–741	4.87e-24
Nimb-2_LVa	896	RT_like super family	cl02808	399–643	4.63e-25
		Exo_endo_phos_2	pfam14529	34–155	3.52e-23
		RVT_1	pfam00078	416–615	2.13e-12
Penelope-1_LVa	808	RT_like	cd00304	459–543	1.20e-08
		GIY-YIG_SF super family	cl15257	691–775	2.31e-10
		RT_G2_intron	cd01651	371–498	1.24e-06
Penelope-3_LVa	826	RT_like	cd00304	480–574	2.68e-09
		GIY-YIG_SF super family	cl15257	752–824	1.61e-07
Penelope-6_LVa	692	GIY-YIG_SF super family	cl15257	615–680	7.08e-09
		RT_like super family	cl02808	362–455	2.54e-06
RTE-1_LVa	746	RT_nLTR_like	cd01650	265–546	3.97e-49
		RVT_1	pfam00078	295–546	2.74e-30
RTE-2_LVa	980	RT_nLTR_like	cd01650	544–797	4.65e-65
		L1-EN	cd09076	47–287	2.06e-36
		RVT_1	pfam00078	555–772	1.93e-32
RTE-3_LVa	1165	RT_nLTR_like	cd01650	735–995	9.97e-54
		L1-EN	cd09076	222–466	4.64e-43
		RVT_1	pfam00078	750–995	1.27e-28

**Fig. 1:**
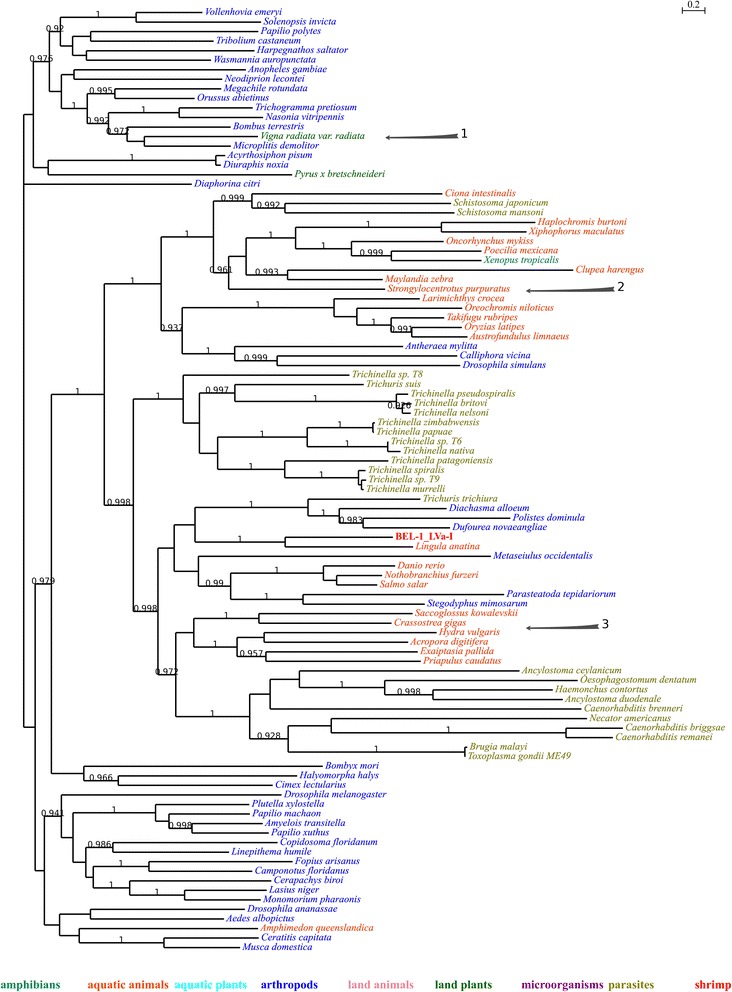
Phylogenetic tree of BEL-1_LVa-I and its homologues. Local support values are only shown for those nodes with support values no less than 0.9. Organism names of respective sequences are colored according to their ecological habit or taxonomy; detailed information of the classified terms could be found in Table 5

**Fig. 2:**
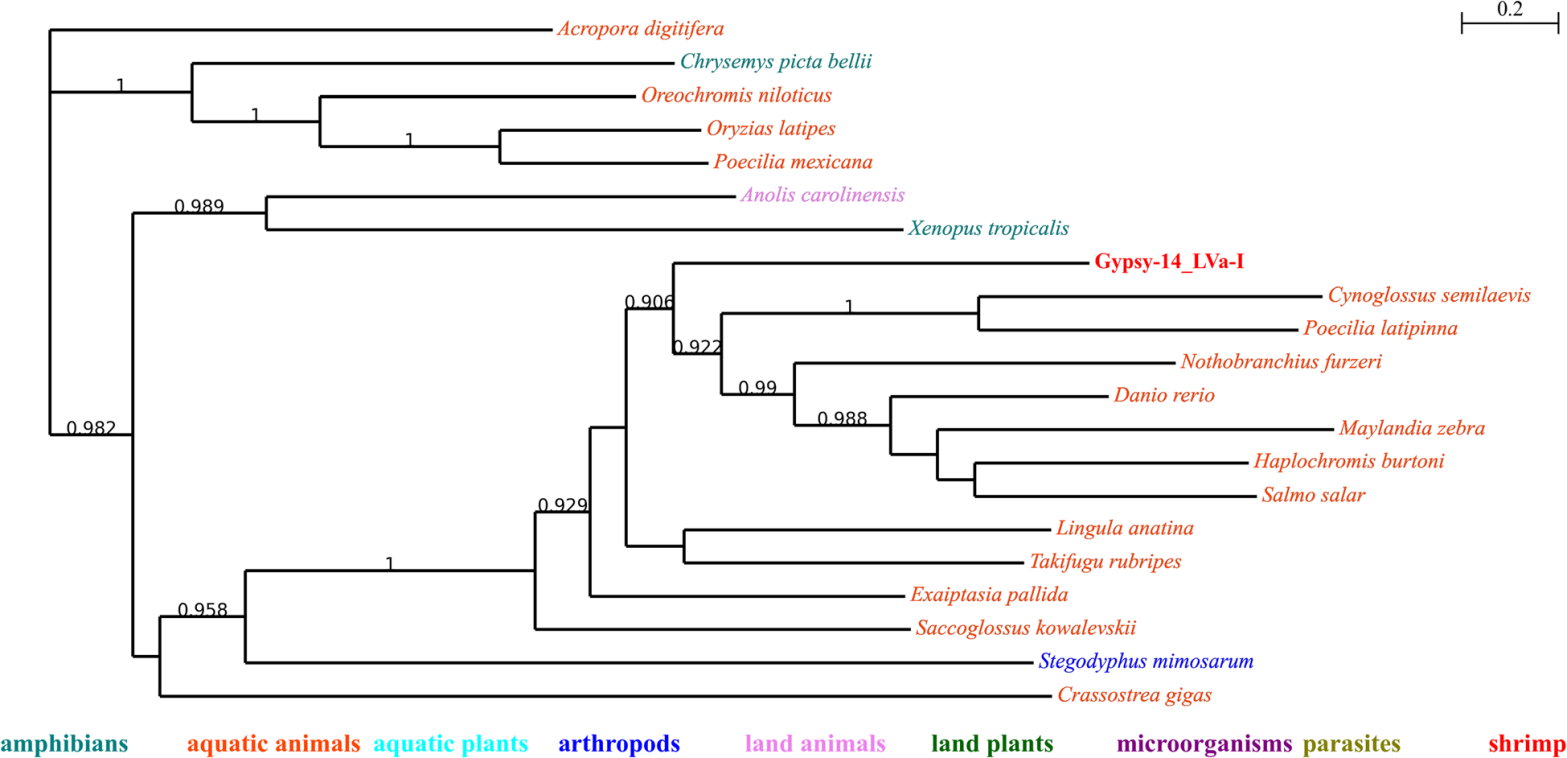
Phylogenetic tree of Gypsy-14_LVa-I and its homologues

**Fig. 3:**
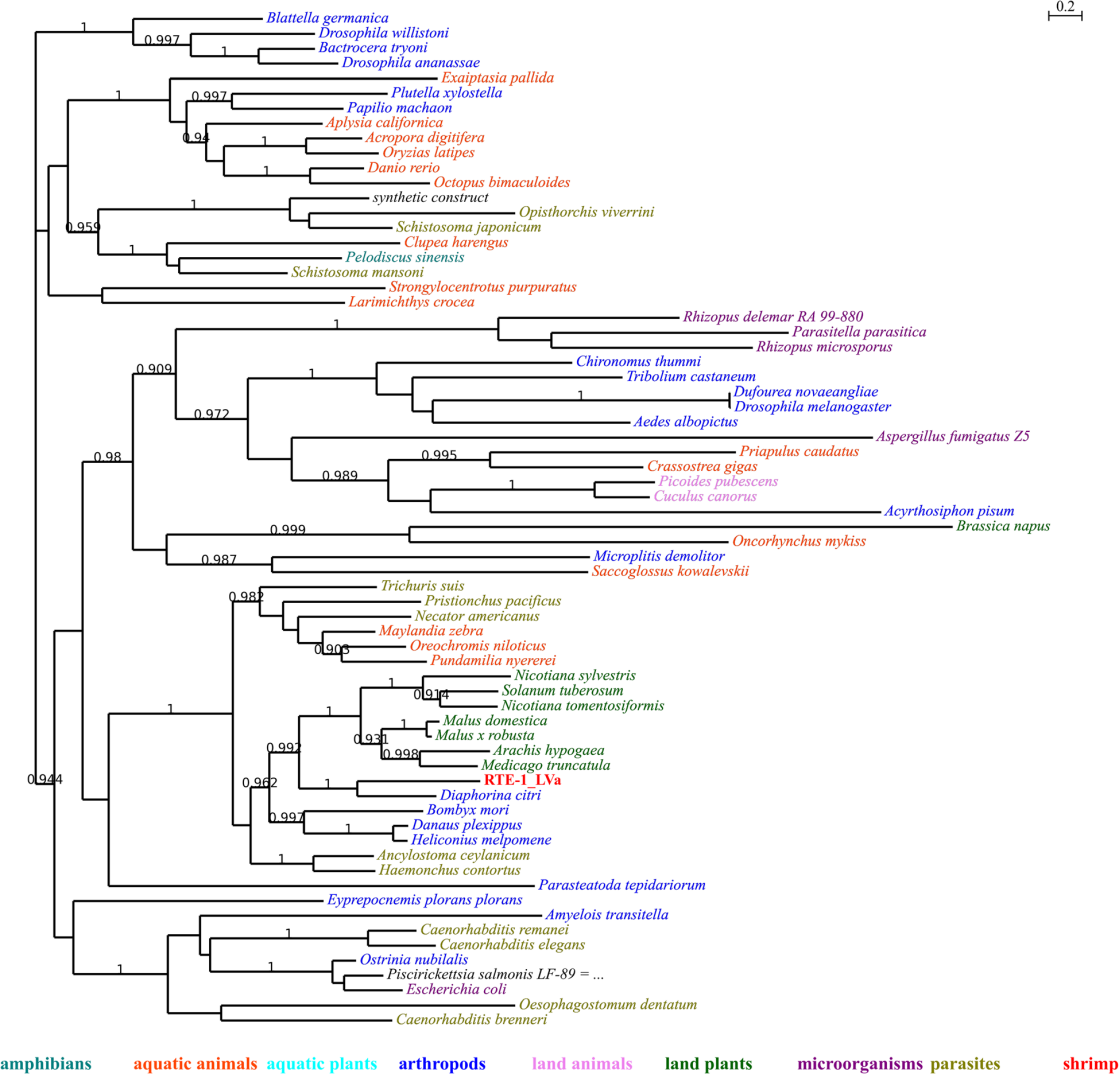
Phylogenetic tree of RTE-1_LVa and its homologues

For example in Fig. 1, many parasites were present in the tree, which indicate that parasitism might play important roles in HTT. Still, it may not be indispensable: in Fig. 2, there is no parasite at all; the close relationship between bees (*Microplitis demolitor* and *Bombus terrestris*) and mung bean (*Vigna radiata var. radiata*) could not be explained by parasitism (indicated by arrow 1 in Fig. 1), either. Aquatic animals tend to group together. However, many of them are actually very distant to each other in evolution (Table 5): purple sea urchin (*Strongylocentrotus purpuratus*) and bony fishes (indicated by arrow 2 in Fig. 1) have diverged for at least 600 million years [28, 29]; *Saccoglossus kowalevskii*, Pacific oyster (*Crassostrea gigas*), hydrozoans (*Hydra vulgaris*), stony corals (*Acropora digitifera*), sea anemones (*Exaiptasia pallida*) and *Priapulus caudatus* also represent a wide range of taxa (indicated by arrow 3 in Fig. 1). For TE families whose closest neighbors were arthropods, they also had relatively close neighbors of distantly related species (Fig. 3 and Additional files 4 and 5), indicating that their homologues might still involve in HTT events. Another point is that microorganisms were also widely present in trees (Additional files 4, 5 and 9). Actually, microorganisms are important donors of horizontally transferred materials found in animals [30]. Here, we conclude that microorganisms and parasites might play similar roles in HTT events: important, yet not indispensable.

**Table 5:** Terms used to distinguish species

	NCBI taxonomy terms
shrimp	Penaeidae
amphibians	Amphibia, Testudines, Annelida, Crocodylia
aquatic animals	Mollusca, Actinopterygii, Echinodermata, Brachiopoda, Enteropneusta, Tunicata, Porifera, Rotifera, Choanoflagellida, Placozoa, Rhizaria, Cyclostomata, Coelacanthiformes, Cnidaria, Euglenozoa, Priapulida, Apusozoa, Heterolobosea, Dipnoi, Chondrichthyes, Cephalochordata
aquatic plants	Viridiplantae (exclude Embryophyta), Haptophyceae, Stramenopiles
arthropods	Arthropoda (exclude Penaeidae)
land animals	Squamata, Mammalia, Aves
land plants	Embryophyta
microorganisms	Bacteria, Fungi, Viruses
parasites	Nematoda, Platyhelminthes, Amoebozoa, Jakobida, Alveolata

Overall speaking, organisms with close ecological relationships tend to group together, even being distantly related in taxonomy. When referring to ecological relationships, we should not overlook the fact that *L. vannamei* and other aquatic species formed a huge food web in water. Therefore, predation among species might greatly facilitate HTTs, either through exchange of parasites and microorganisms, or through direct flow of DNA. After all, naked DNA and RNA can circulate in animal bodily fluids [31]. The huge amounts of TEs may also ensure their success of passing through a digestive system and other barriers.

It has been proposed that HTTs among plants might provide an escape route from silencing and elimination and are thus essential for TEs’ survival in plants [6]. Yet on the other hand, the acquisition of foreign genes by horizontal transfer may enhance the evolutionary potential of the recipient lineage [12]. Although the expansions of TEs look like selfish and parasitic, TEs are actually important drivers of genome evolution: they can provide raw material for novel genes and contribute to regulation and generation of allelic diversity [14, 32, 33]. In this study, the frequent exchange of TEs between *L. vannamei* and other aquatic species may also provide some evolutionary advantages for them.

### HTT involved transcripts might play important roles in antiviral immunity

**Fig. 4:**
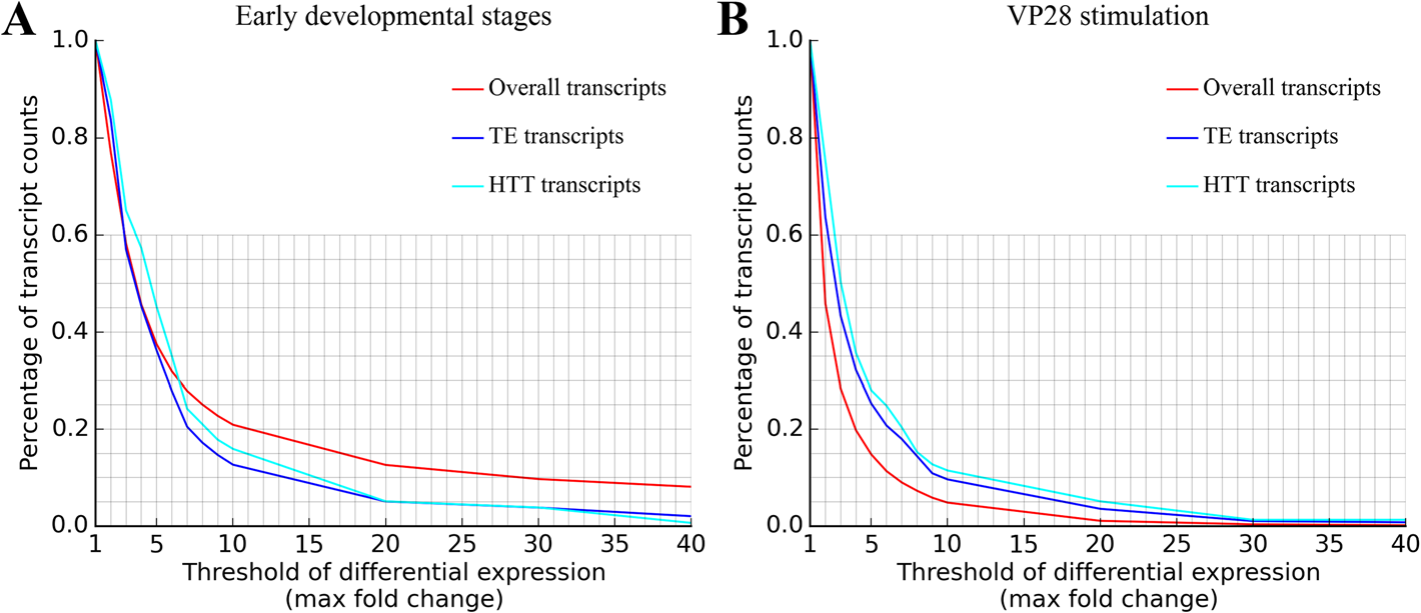
Expression profile of overall transcripts, TE transcripts and HTT transcripts. Raw sequencing reads of two NCBI BioProjects were aligned and counted: transcriptome of five early stages in *L. vannamei* (**a**) and haemocyte transcriptome of *L. vannamei* after the successive stimulation of recombinant VP28 (**b**). The threshold of differential expression represents the max fold change of transcript read counts among different experimental groups

To elucidate whether TEs, especially TEs involved in HTT events, have any biological functions, we analyzed the expression level of all transcripts in two NCBI BioProjects: (i) transcriptome of five early stages in *L. vannamei*, namely embryo, nauplius, zoe, mysis and postlarvae; and (ii) haemocyte transcriptome of *L. vannamei* after the successive stimulation of recombinant VP28. VP28 is known as one of the major envelope proteins of WSSV and is likely to play a key role in the initial steps of the systemic WSSV infection in shrimp [34]. As shown in Fig. 4, TE/HTT and overall transcripts showed different expression patterns in both BioProjects : in early developmental stages, the proportion of differentially expressed TE/HTT transcripts is generally lower than that of overall transcripts (Fig. 4a) ; while in response to VP28 stimulation, the proportion of differentially expressed TE/HTT transcripts is consistently higher than that of overall transcripts (Fig. 4b). Evidently, even TE/HTT transcripts may have some roles in early development, their effects would be diluted in overall transcripts; on the other hand, their possible roles in antiviral immunity are likely to be enriched. Using One-Class Support Vector Machines (SVM) models [35, 36], we predicted transcripts that showed similar expression pattern to HTT transcripts in both BioProjects. During early developmental stages, nine transcripts showed similar expression pattern to HTT transcripts; however, none of them have significant blastx hits (*E*-value < 1e-5), making it impossible to deduce their possible functions. Under VP28 stimulation, 34 transcripts showed similar expression pattern to HTT transcripts, of which seven have significant blastx hits with ascertained biological functions (Table 6). Transcripts listed in Table 6 (except the last one) are not likely to be direct immune genes, yet their fundamental roles must be indispensable in antiviral immunity (and in other biotic stresses) [37].

**Table 6:** Transcripts that showed similar expression pattern to HTT transcripts under VP28 stimulation

ID	Blastx hits	Function	*E*-value
comp40618_c1_seq3	Methyl-CpG-binding domain protein 1	Transcriptional repressor	1.36e-10
comp40966_c0_seq1	Apolipophorins-like protein	Transporter of various types of lipids in hemolymph	3.42e-20
comp43253_c1_seq1	Rhophilin-2	Signal transduction in Rho pathway	0.0
comp45420_c0_seq15	LIM and calponin domains-containing protein	Actomyosin structure organization	3.93e-37
comp45457_c1_seq9	Open rectifier potassium channel protein	Background potassium channel	2.97e-27
comp2416014_c0_seq1	Proto-oncogene tyrosine-protein kinase ROS	Epithelial cell differentiation	1.01e-37
comp6562829_c0_seq1	Linear gramicidin synthase subunit B	Antibiotic biosynthetic process	1.19e-07

**Fig. 5:**
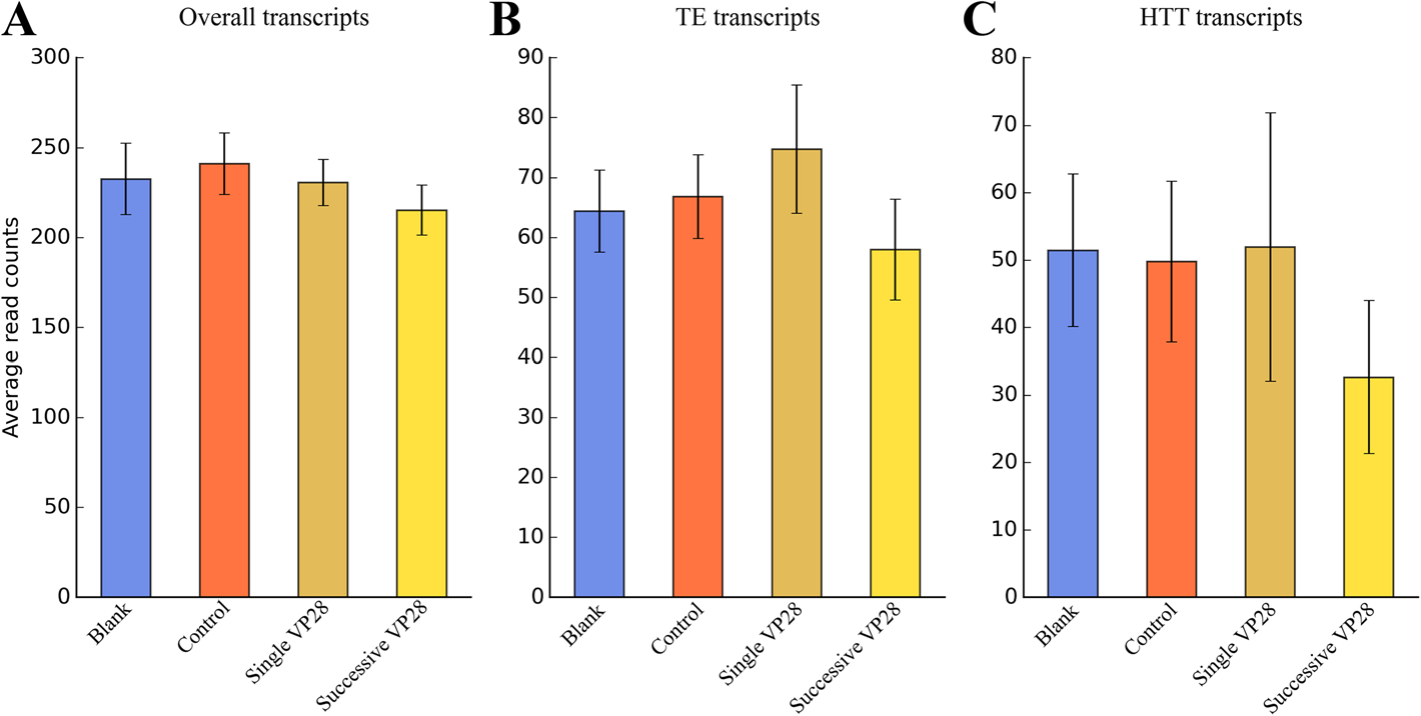
Average read counts of transcripts in different experimental groups. The BioProject is the haemocyte transcriptome of *L. vannamei* after the successive stimulation of recombinant VP28. The threshold of differential expression is 1, which indicates that whole collection of overall transcripts (**a**), TE transcripts (**b**) and HTT transcripts (**c**) are included. Error bars represent SE; no significant difference exists between any two groups (*P* > 0.05, determined by one-way ANOVA)

**Fig. 6:**
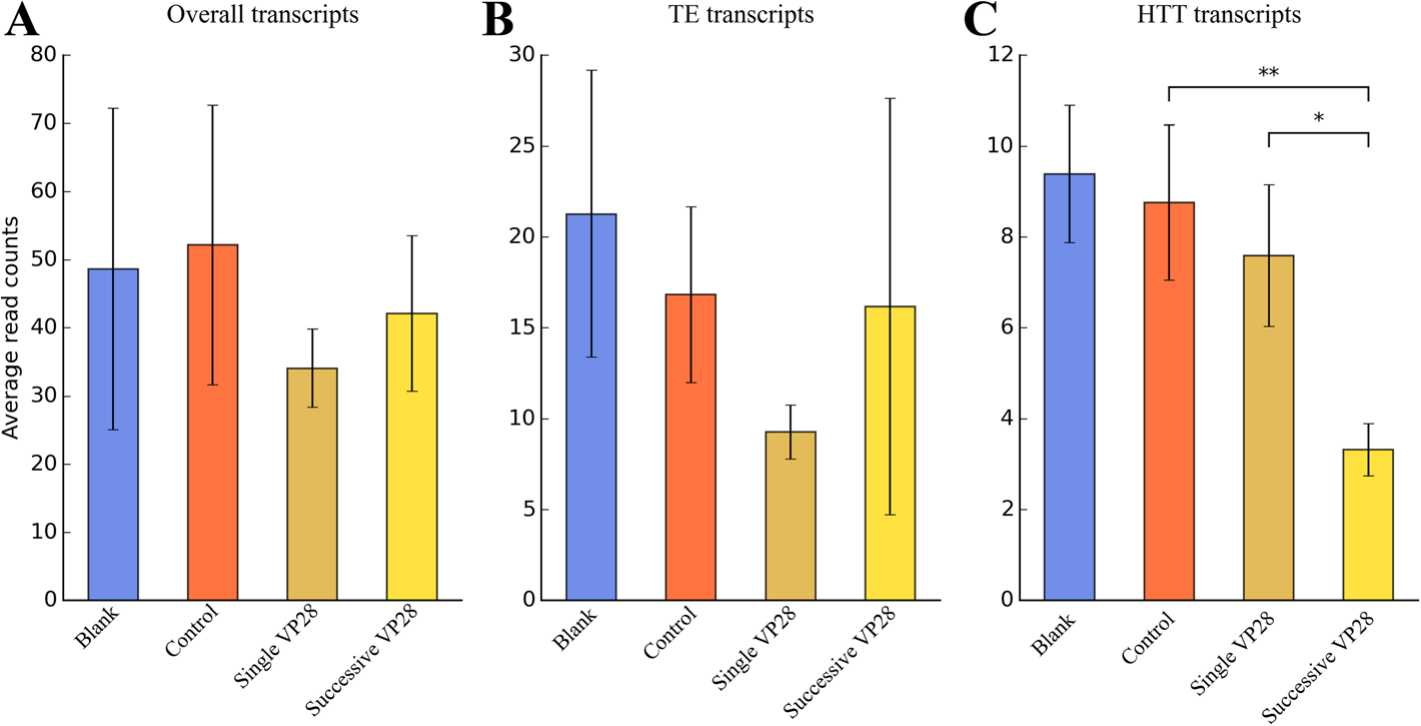
Average read counts of differentially expressed transcripts in different experimental groups. The BioProject is also the haemocyte transcriptome of *L. vannamei* after the successive stimulation of recombinant VP28. The threshold of differential expression is 6, therefore around 11 % of overall transcripts (**a**), 20 % of TE transcripts (**b**) and 25 % of HTT transcripts (**c**) are included (as indicated in Fig. 4). Two asterisks represent very significant difference (**P < 0.01, determined by one-way ANOVA) between mean values of two groups, while one asterisk represents significant difference (*P < 0.05); error bars represent SE

The injection of VP28 into shrimp has been proved to increase their resistance to invasive WSSV [38]. GO enrichment analysis (BioProject: PRJNA233549) indicated that the successive VP28 stimulation could modulate cytoskeleton integration and redox to promote the phagocytosis activity of shrimp haemocytes [38]. Apart from up-regulation of antiviral genes, the down-regulation of some other functional genes may also be helpful. For example, the small GTP-binding protein Rab7 (GenBank ID: FJ811529.1) is a VP28-binding protein [39]. Injection of VP28 down-regulated the expression of Rab7 gene (Additional file 11), which is in accordance with previous finding that suppression of Rab7 inhibits WSSV (and also yellow head virus, YHV) infection in shrimp [40]. To elucidate more exact roles of TE/HTT transcripts, we further analyzed the expression level of overall/TE/HTT transcripts in different experimental groups: blank (no treatment), control (two injections of PBS buffer), single VP28 (one injection of PBS buffer and one injection of VP28) and successive VP28 (two injections of VP28) [38]. Two thresholds of differential expression were selected: at the threshold of 1, the whole collection of a transcript set (overall, TE or HTT) will be included; at the threshold of 6, it means the max fold change of any transcript among different experimental groups exceeds 6. At the threshold of 1, the mean values of expression levels varied, but no statistical significance (*P* < 0.05) was found in any transcript set. This is in accordance with the hypothesis that most genes are not differentially expressed [41] (Fig. 5). At the threshold of 6, on the other hand, the expression level of HTT transcripts in successive VP28 group was significantly lower than other groups (Fig. 6). Furthermore, at the threshold of 6, there are 39 HTT transcripts, seven of which contain fragments of WSSV (as described above in section 2 of Results and discussion, also see Additional file 12). Taken together, we suggest that the down-regulation of HTT transcripts in VP28 stimulation is not likely to be an incidental or side effect, but reflect their potential inhibitory roles in antiviral immunity.

## Conclusions

Although the number of presumptive horizontally transferred genes is increasing, the exact role of HT/HTT in the evolution of unicellular eukaryotes is still blurry. Our knowledge about the underlying mechanism is even more limited. In this study, we found that in *L. vannamei*, an ancient crustacean, a considerable number of transcripts are also involved in HTT events. Nearly all of the HTT transcripts are transcripts of retrotransposons, which is in accordance with previous findings. Phylogenetic analyses revealed that *L. vannamei* TEs are often most close to TEs from aquatic species. Furthermore, TEs from other aquatic species, the taxonomic relationship among which are often very far away, also tend to group together. We suggest that HTT events might frequently occur among species that have close ecological relationships, the underlying impetus of which might be predation among those species. Through analyses of expression profile, we found that TE/HTT transcripts are more likely to play important roles in antiviral immunity, and they might actually act as inhibitors of antiviral immunity.

## Methods

### Identification of transcripts derived from TEs

A new transcriptome assembly of *L. vannamei* was downloaded from http://oaktrust.library.tamu.edu/handle/1969.1/152151, which contains 110,474 contigs with an N50 of 2701 bases [18]. Each assembled contig was viewed as a transcript, regardless of alternative transcripts that share the same precursors. To exclude artifacts [42] and possible contaminations in sampling, these transcripts were conducted local blastn search against whole collection of *L. vannamei* sequences downloaded from NCBI. Fifty-six thousand six hundred eight transcripts with higher similarities to already existed *L. vannamei* nucleotide sequences or expressed sequence tags (ESTs) were selected for further analysis (for more details, see Additional file 13). To isolate TE related transcripts, we conducted a local BLAST based two-step searching of similar domains/sequences. First, the fifty-six thousand six hundred transcripts were conducted blastx search against cdd_delta [43], which contains 26,482 conserved domain sequences downloaded from ftp://ftp.ncbi.nlm.nih.gov/blast/db/. 813 transcripts were identified as TE-related because each of them has at least one hit that is TE-related (has the character string ‘transposon’ in sequence description). Second, to exclude transcripts that are actually transcripts of single/low copy genes that happened to contain TE-related domain(s), two further sequence searches were conducted for the above 813 transcripts: (i) blastx again cdd_delta again, and (ii) tblastx against a database contains 45,725 repetitive sequences downloaded from Repbase Update (http://www.girinst.org/, relase 20.09) [44]. The criteria here were as follows: for a given query transcript, the *E*-value of the top hit in tblastx should be lower than 1e-5 and also lower than that in blastx top hit. Finally, 395 transcripts were identified as transcripts of TEs, with very high reliability.

### Characterization of superfamilies and families of TE derived transcripts

The 395 TE derived transcripts were conducted tblastx to determine their superfamilies and blastn to determine their families. The database used here is the same as the one described above which contains 45,725 repetitive sequences. Briefly, a transcript was thought belonging to the same superfamily as its top hit in tblastx results; to determine its family classification, the top hit in blastn results should come from *L. vannamei* and meet an *E*-value cut-off at 1e-20. Therefore, 376 transcripts had their superfamilies determined while only 230 transcripts could be identified as transcripts of already known *L. vannamei* TE families. In total, 31 families were identified and only two were not consistent with identified superfamilies.

### Evidence of HTTs and identification of *L. vannamei* TE families involved in HTTs

A Biopython [45] module, Bio.Blast.NCBIWWW, was used to query the NCBI BLAST Nucleotide (nt) database over the Internet using the 395 TE derived transcripts. All hits with *E*-value lower than 1e-5 were screened for their taxa. To effectively distinguish the organisms in the hits, 17 taxa were selected (as shown in Table 2). Their frequencies as top hit were counted. Since penaeidae shrimps are very close in evolution [46], they were excluded from the taxon arthropoda, that is to say hits from penaeidae family were filtered (mainly *L. vannamei*, *Penaeus monodon* and *Marsupenaeus japonicus*). Transcripts that showed highest sequence similarity to distantly related taxa, which meant the top hits were not from arthropods, were believed to be involved in HTTs. If the corresponding families of those transcripts were from *L. vannamei*, then they will be isolated. In total, 16 *L. vannamei* TE families were possibly involved in HTTs, representing 83 transcripts.

### Presence of HTT-involved *L. vannamei* TE families’ homologues in other species

The Bio.Blast.NCBIWWW module was also used for the 16 *L. vannamei* TE families to conducted homology search against the NCBI BLAST chromosome and HTGS (high throughput genomic sequences) databases, respectively. The threshold of *E*-value was set to be 1e-10. For a given TE family, its best hit in searching against the two databases were extracted, the taxon and organism of which was also screened as described above.

### Phylogenetic analyses

Of the 16 *L. vannamei* TE families, 10 have coding regions (CDS) being annotated. Therefore, the longest protein sequence (in case there are more than one CDS) of each TE family was extracted and combined. The conserved domains within these protein sequences were predicated by the NCBI online tool CDD search (http://www.ncbi.nlm.nih.gov/Structure/cdd/wrpsb.cgi) and the results are displayed in Table 4. These protein sequences were used to conduct blastp search against NCBI BLAST Protein (nr) database. The threshold of *E*-value was set to be 1e-20; however, the actual *E*-value of all significant hits was 0. To remove redundancies, hits of one given query sequence were selected in the following way: hits with query length coverage less than 60 % were abandoned; the organisms of remaining hits were screened and only the top hit from the same organism was selected for further analyses (Additional file 14). The selected protein sequences were all downloaded from NCBI using Batch Entrez. All sequences, including queries, were aligned with MUSCLE [47]. We used FastTree [26] and RAxML [27] to construct phylogenetic trees from the multiple alignments (Additional file 15). FastTree trees were built using the defaulted JTT + CAT model and gamma approximation on substitution rates. RAxML trees were built using LG model (selected by automatic test of all models), gamma approximation on substitution rates and 100 bootstraps. Approximately unbiased (AU) tests of RAxML tree topologies were carried out using CONSEL [48].

### Identification of differentially expressed transcripts

Raw sequencing data of two NCBI BioProjects, PRJNA253518 and PRJNA233549, were downloaded from NCBI ftp site (ftp://ftp.ncbi.nlm.nih.gov/) (Additional file 16). The project PRJNA253518 is transcriptome of five early stages in *L. vannamei*, namely embryo, nauplius, zoe, mysis and postlarvae. The project PRJNA233549 is haemocyte transcriptome of *L. vannamei* after the successive stimulation of recombinant VP28 [38]. To find transcripts differentially expressed in different circumstances, those fifty-six thousand six hundred transcripts were conducted alignments against reads from the two projects, using the Burrows-Wheeler Alignment tool (BWA, version 0.7.5a) [49]. The number of unambiguously matched reads to each transcript was counted using the HTSeq framework [50]. These counts were then normalized by edgeR [41, 51] for subsequent differential expression analysis. We set a range of values (1 to 40) as thresholds to indicate the degree of differential expression. Briefly, the read counts (represent expression levels) of one specific transcript in different experimental groups are usually different and should have a maximum count and a minimum count (if this is 0, then a pseudocount of one will be added). The max fold change of one transcript in a BioProject is calculated as below:

$$ \max \mathrm{fold}\;\mathrm{change} = \mathrm{maximum}\ \mathrm{count}/\mathrm{minimum}\ \mathrm{count} $$

Naturally, at the threshold of 1, all transcripts will be included; while at the threshold of 10, only 20 % or fewer transcripts will be included (see Fig. 4).

To predict transcripts of functional genes (other than TEs) that showed similar expression pattern to HTT transcripts, we developed One-Class SVM models [35] implemented in Scikt-learn [36], a Python module for machine learning. The defaulted RBF kernel was chosen. HTT transcripts with max fold change above four (in order to get more than 50 samples) in either BioProject were selected as training data. Transcripts that predicted to be positive were collected and used to conduct blastx search against NCBI BLAST Protein (nr) database.

## Additional files

Additional file 1: Detailed information of identified TE transcripts. (DOCX 72.2 kb)
Additional file 2: Longest protein sequences of 10 *L. vannamei* TE families. (FASTA 10 kb)
Additional file 3: Phylogenetic trees built by RAxML. (DOCX 3871 kb)
Additional file 4: Phylogenetic tree of Nimb-1_LVa and its homologues. (PNG 2283 kb)
Additional file 5: Phylogenetic tree of Nimb-2_LVa and its homologues. (PNG 1345 kb)
Additional file 6: Phylogenetic tree of Penelope-1_LVa and its homologues. (PNG 560 kb)
Additional file 7: Phylogenetic tree of Penelope-3_LVa and its homologues. (PNG 535 kb)
Additional file 8: Phylogenetic tree of Penelope-6_LVa and its homologues. (PNG 854 kb)
Additional file 9: Phylogenetic tree of RTE-2_LVa and its homologues. (PNG 2080 kb)
Additional file 10: Phylogenetic tree of RTE-3_LVa and its homologues. (PNG 1074 kb)
Additional file 11: Expression change of Rab7 gene in response to VP28 stimulation. (DOCX 16 kb)
Additional file 12: HTT transcripts’ expression change in two BioProjects. (XLSX 24 kb)
Additional file 13: Supplementary methods and source codes. (DOCX 72 kb)
Additional file 14: GenBank accession numbers of protein sequences used for phylogenetic analyses. (XLSX 20 kb)
Additional file 15: Multiple sequence alignments used for phylogenetic analyses. (ZIP 546 kb)
Additional file 16: Detailed information of two NCBI BioProjects, PRJNA253518 and PRJNA233549. (XLSX 29 kb)

## References

[1] Zhaxybayeva O, Doolittle WF (2011). Lateral gene transfer. Curr Biol.

[2] Ochman H, Lawrence JG, Groisman EA (2000). Lateral gene transfer and the nature of bacterial innovation. Nature.

[3] Skippington E, Ragan MA (2011). Lateral genetic transfer and the construction of genetic exchange communities. FEMS Microbiol Rev.

[4] Chapman JA, Kirkness EF, Simakov O, Hampson SE, Mitros T, Weinmaier T, Rattei T, Balasubramanian PG, Borman J, Busam D (2010). The dynamic genome of Hydra. Nature.

[5] Danchin EGJ, Rosso MN, Vieira P, de Almeida-Engler J, Coutinho PM, Henrissat B, Abad P (2010). Multiple lateral gene transfers and duplications have promoted plant parasitism ability in nematodes. P Natl Acad Sci USA.

[6] El Baidouri M, Carpentier MC, Cooke R, Gao D, Lasserre E, Llauro C, Mirouze M, Picault N, Jackson SA, Panaud O (2014). Widespread and frequent horizontal transfers of transposable elements in plants. Genome Res.

[7] Gladyshev EA, Meselson M, Arkhipova IR (2008). Massive horizontal gene transfer in bdelloid rotifers. Science.

[8] Graham LA, Lougheed SC, Ewart KV, Davies PL (2008). Lateral Transfer of a Lectin-Like Antifreeze Protein Gene in Fishes. PLoS ONE.

[9] Hotopp JCD, Clark ME, Oliveira DCSG, Foster JM, Fischer P, Munoz Torres MC, Giebel JD, Kumar N, Ishmael N, Wang S (2007). Widespread lateral gene transfer from intracellular bacteria to multicellular eukaryotes. Science.

[10] Rot C, Goldfarb I, Ilan M, Huchon D. Putative cross-kingdom horizontal gene transfer in sponge (Porifera) mitochondria. BMC Evol Biol. 2006;6(71). 10.1186/1471-2148-6-71 PMC1618405 16972986

[11] Walsh AM, Kortschak RD, Gardner MG, Bertozzi T, Adelson DL (2013). Widespread horizontal transfer of retrotransposons. P Natl Acad Sci USA.

[12] Wijayawardena BK, Minchella DJ, DeWoody JA (2013). Hosts, parasites, and horizontal gene transfer. Trends Parasitol.

[13] Wicker T, Sabot F, Hua-Van A, Bennetzen JL, Capy P, Chalhoub B, Flavell A, Leroy P, Morgante M, Panaud O (2007). A unified classification system for eukaryotic transposable elements. Nat Rev Genet.

[14] Kazazian HH (2004). Mobile Elements: Drivers of Genome Evolution. Science.

[15] Kumar A, Jeffrey B (1999). Plant retrotransposons. Annu Rev Genet.

[16] Boissinot S, Chevret P, Furano AV (2000). L1 (LINE-1) retrotransposon evolution and amplification in recent human history. Mol Biol Evol.

[17] Piednoël M, Donnart T, Esnault C, Graça P, Higuet D, Bonnivard E (2013). LTR-Retrotransposons in *R. exoculata* and Other Crustaceans: The Outstanding Success of GalEa-Like Copia Elements. PLoS ONE.

[18] Ghaffari N, Sanchez-Flores A, Doan R, Garcia-Orozco KD, Chen PL, Ochoa-Leyva A, Lopez-Zavala AA, Carrasco JS, Hong C, Brieba LG (2014). Novel transcriptome assembly and improved annotation of the whiteleg shrimp (*Litopenaeus vannamei*), a dominant crustacean in global seafood mariculture. Sci Rep-UK.

[19] Li J, Li J, Chen P, Liu P, He Y (2015). Transcriptome analysis of eyestalk and hemocytes in the ridgetail white prawn *Exopalaemon carinicauda*: assembly, Annotation and Marker Discovery. Mol Biol Rep.

[20] Shen H, Hu Y, Ma Y, Zhou X, Xu Z, Shui Y, Li C, Xu P, Sun X (2014). In-Depth Transcriptome Analysis of the Red Swamp Crayfish *Procambarus clarkii*. PLoS ONE.

[21] Chow S, Dougherty WJ, Sandifer PA (1990). Meiotic chromosome complements and nuclear DNA contents of four species of shrimps of the genus *Penaeus*. J Crustacean Biol.

[22] Sookruksawong S, Sun F, Liu Z, Tassanakajon A (2013). RNA-Seq analysis reveals genes associated with resistance to Taura syndrome virus (TSV) in the Pacific white shrimp *Litopenaeus vannamei*. Dev Comp Immunol.

[23] Pradeep B, Shekar M, Karunasagar I, Karunasagar I (2008). Characterization of variable genomic regions of Indian white spot syndrome virus. Virology.

[24] Thitamadee S, Prachumwat A, Srisala J, Jaroenlak P, Salachan PV, Sritunyalucksana K, Flegel TW, Itsathitphaisarn O (2016). Review of current disease threats for cultivated penaeid shrimp in Asia. Aquaculture.

[25] Koski LB, Golding GB (2001). The Closest BLAST Hit Is Often Not the Nearest Neighbor. J Mol Evol.

[26] Price MN, Dehal PS, Arkin AP (2010). FastTree 2—Approximately Maximum-Likelihood Trees for Large Alignments. PLoS ONE.

[27] Stamatakis A (2014). RAxML version 8: a tool for phylogenetic analysis and post-analysis of large phylogenies. Bioinformatics.

[28] Hedges SB, Marin J, Suleski M, Paymer M, Kumar S (2015). Tree of Life Reveals Clock-Like Speciation and Diversification. Mol Biol Evol.

[29] Peterson KJ, Cotton JA, Gehling JG, Pisani D (2008). The Ediacaran emergence of bilaterians: congruence between the genetic and the geological fossil records. Philo Trans R Soc B Biol Sci.

[30] Boto L. Horizontal gene transfer in the acquisition of novel traits by metazoans. P Roy Soc B-Biol Sci. 2014;281(20132450). 10.1098/rspb.2013.2450 PMC3896011 24403327

[31] Stroun M, Lyautey J, Lederrey C, Mulcahy HE, Anker P (2001). Alu repeat sequences are present in increased proportions compared to a unique gene in plasma/serum DNA: evidence for a preferential release from viable cells?. Ann NY Acad Sci.

[32] Abrusan G, Szilagyi A, Zhang Y, Papp B (2013). Turning gold into ‘junk’: transposable elements utilize central proteins of cellular networks. Nucleic Acids Res.

[33] Nefedova LN, Kuzmin IV, Makhnovskii PA, Kim AI (2014). Domesticated retroviral GAG gene in Drosophila: New functions for an old gene. Virology.

[34] van Hulten MCW, Witteveldt J, Snippe M, Vlak JM (2001). White spot syndrome virus envelope protein VP28 is involved in the systemic infection of shrimp. Virology.

[35] Schölkopf B, Platt JC, Shawe-Taylor J, Smola AJ, Williamson RC (2001). Estimating the support of a high-dimensional distribution. Neural Comput.

[36] Pedregosa F, Varoquaux G, Gramfort A, Michel V, Thirion B, Grisel O, Blondel M, Prettenhofer P, Weiss R, Dubourg V (2011). Scikit-learn: Machine Learning in Python. J Mach Learn Res.

[37] Liu H, Söderhäll K, Jiravanichpaisal P (2009). Antiviral immunity in crustaceans. Fish Shellfish Immunol.

[38] Wang L, Sun X, Zhou Z, Zhang T, Yi Q, Liu R, Wang M, Song L (2014). The promotion of cytoskeleton integration and redox in the haemocyte of shrimp *Litopenaeus vannamei* after the successive stimulation of recombinant VP28. Dev Comp Immunol.

[39] Sritunyalucksana K, Wannapapho W, Lo CF, Flegel TW (2006). PmRab7 is a VP28-binding protein involved in white spot syndrome virus infection in shrimp. J Virol.

[40] Ongvarrasopone C, Chanasakulniyom M, Sritunyalucksana K, Panyim S (2008). Suppression of PmRab7 by dsRNA inhibits WSSV or YHV infection in shrimp. Mar Biotechnol.

[41] Dillies MA, Rau A, Aubert J, Hennequet-Antier C, Jeanmougin M, Servant N, Keime C, Marot G, Castel D, Estelle J (2013). A comprehensive evaluation of normalization methods for Illumina high-throughput RNA sequencing data analysis. Brief Bioinform.

[42] Birney E (2011). Assemblies: the good, the bad, the ugly. Nat Methods.

[43] Marchler-Bauer A, Derbyshire MK, Gonzales NR, Lu S, Chitsaz F, Geer LY, Geer RC, He J, Gwadz M, Hurwitz DI (2015). CDD: NCBI’s conserved domain database. Nucleic Acids Res.

[44] Bao W, Kojima KK, Kohany O (2015). Repbase Update, a database of repetitive elements in eukaryotic genomes. Mobile DNA-UK.

[45] Cock PJA, Antao T, Chang JT, Chapman BA, Cox CJ, Dalke A, Friedberg I, Hamelryck T, Kauff F, Wilczynski B (2009). Biopython: freely available Python tools for computational molecular biology and bioinformatics. Bioinformatics.

[46] Ma KY, Chan TY, Chu KH (2009). Phylogeny of penaeoid shrimps (Decapoda: Penaeoidea) inferred from nuclear protein-coding genes. Mol Phylogenet Evol.

[47] Edgar RC (2004). MUSCLE: multiple sequence alignment with high accuracy and high throughput. Nucleic Acids Res.

[48] Shimodaira H, Hasegawa M (2001). CONSEL: for assessing the confidence of phylogenetic tree selection. Bioinformatics.

[49] Li H, Durbin R (2009). Fast and accurate short read alignment with Burrows-Wheeler transform. Bioinformatics.

[50] Anders S, Pyl PT, Huber W (2015). HTSeq—a Python framework to work with high-throughput sequencing data. Bioinformatics.

[51] Robinson MD, McCarthy DJ, Smyth GK (2009). edgeR: a Bioconductor package for differential expression analysis of digital gene expression data. Bioinformatics.

