# Supplementary material for: Close ecological relationship among species facilitated horizontal transfer of retrotransposons
Source: BMC Evol Biol. 2016 Oct 7;16:201. doi: 10.1186/s12862-016-0767-0 (PMC5055719; doi:10.1186/s12862-016-0767-0)
Supplement: Additional file 1: — Detailed information of identified TE transcripts. (DOCX 72.2 kb) [file 12862_2016_767_MOESM1_ESM.docx]

Detailed information of projects PRJNA253518 and PRJNA233549

| BioProject | Library information | SRA experiments and download links |
| --- | --- | --- |
| PRJNA253518 | Instrument: Illumina HiSeq 2000  Strategy: RNA-Seq  Source: TRANSCRIPTOMIC  Selection: PolyA  Layout: SINGLE | postlarvae stage of Litopenaeus vannamei  1 ILLUMINA (Illumina HiSeq 2000) run: 25.8M spots, 2.3G bases, 1.6Gb downloads  Accession: SRX625475 (SRR1460505.sra)  ftp.ncbi.nlm.nih.gov/sra/sra-instant/reads/ByExp/sra/SRX/SRX625/SRX625475/SRR1460505/  mysis stage of Litopenaeus vannamei  1 ILLUMINA (Illumina HiSeq 2000) run: 27M spots, 2.4G bases, 1.6Gb downloads  Accession: SRX625474 (SRR1460504.sra)  ftp.ncbi.nlm.nih.gov/sra/sra-instant/reads/ByExp/sra/SRX/SRX625/SRX625474/SRR1460504/  zoea stage of Litopenaeus vannamei  1 ILLUMINA (Illumina HiSeq 2000) run: 26.7M spots, 2.4G bases, 1.6Gb downloads  Accession: SRX625467 (SRR1460495.sra)  ftp.ncbi.nlm.nih.gov/sra/sra-instant/reads/ByExp/sra/SRX/SRX625/SRX625467/SRR1460495/  nauplius stage of Litopenaeus vannamei  1 ILLUMINA (Illumina HiSeq 2000) run: 26.4M spots, 2.4G bases, 1.5Gb downloads  Accession: SRX625466 (SRR1460494.sra)  ftp.ncbi.nlm.nih.gov/sra/sra-instant/reads/ByExp/sra/SRX/SRX625/SRX625466/SRR1460494/  embryo stage of Litopenaeus vannamei  1 ILLUMINA (Illumina HiSeq 2000) run: 25.8M spots, 2.3G bases, 1.4Gb downloads  Accession: SRX625463 (SRR1460493.sra)  ftp.ncbi.nlm.nih.gov/sra/sra-instant/reads/ByExp/sra/SRX/SRX625/SRX625463/SRR1460493/ |
| PRJNA233549 | Instrument: AB SOLiD 4 System  Strategy: RNA-Seq  Source: TRANSCRIPTOMIC  Selection: cDNA  Layout: SINGLE | Shrimp haemocyte transcriptome after the successive VP28 stimulation  2 ABI_SOLID (AB SOLiD 4 System) runs: 66.6M spots, 3.3G bases, 2.7Gb downloads  Accession: SRX423251 (SRR1104086.sra SRR1104087.sra)  ftp.ncbi.nlm.nih.gov/sra/sra-instant/reads/ByExp/sra/SRX/SRX423/SRX423251/SRR1104086/  /sra/sra-instant/reads/ByExp/sra/SRX/SRX423/SRX423251/SRR1104087/  Shrimp haemocyte transcriptome after the single VP28 stimulation  2 ABI_SOLID (AB SOLiD 4 System) runs: 63.1M spots, 3.2G bases, 2.5Gb downloads  Accession: SRX423248 (SRR1104084.sra SRR1104085.sra)  /sra/sra-instant/reads/ByExp/sra/SRX/SRX423/SRX423248/SRR1104084/  /sra/sra-instant/reads/ByExp/sra/SRX/SRX423/SRX423248/SRR1104085/  Shrimp haemocyte transcriptome after PBS stimulation  2 ABI_SOLID (AB SOLiD 4 System) runs: 55.2M spots, 2.8G bases, 2.2Gb downloads  Accession: SRX423246 (SRR1104080.sra SRR1104083.sra)  /sra/sra-instant/reads/ByExp/sra/SRX/SRX423/SRX423246/SRR1104080/  /sra/sra-instant/reads/ByExp/sra/SRX/SRX423/SRX423246/SRR1104083/  Shrimp haemocyte transcriptome under control condition  2 ABI_SOLID (AB SOLiD 4 System) runs: 59M spots, 3G bases, 2.4Gb downloads  Accession: SRX422747 (SRR1104081.sra SRR1104082.sra)  /sra/sra-instant/reads/ByExp/sra/SRX/SRX422/SRX422747/SRR1104081/  /sra/sra-instant/reads/ByExp/sra/SRX/SRX422/SRX422747/SRR1104082/ |
