# Supplementary material for: Close ecological relationship among species facilitated horizontal transfer of retrotransposons
Source: BMC Evol Biol. 2016 Oct 7;16:201. doi: 10.1186/s12862-016-0767-0 (PMC5055719; doi:10.1186/s12862-016-0767-0)
Supplement: Additional file 3: — Phylogenetic trees built by RAxML. (DOCX 3871 kb) [file 12862_2016_767_MOESM3_ESM.docx]

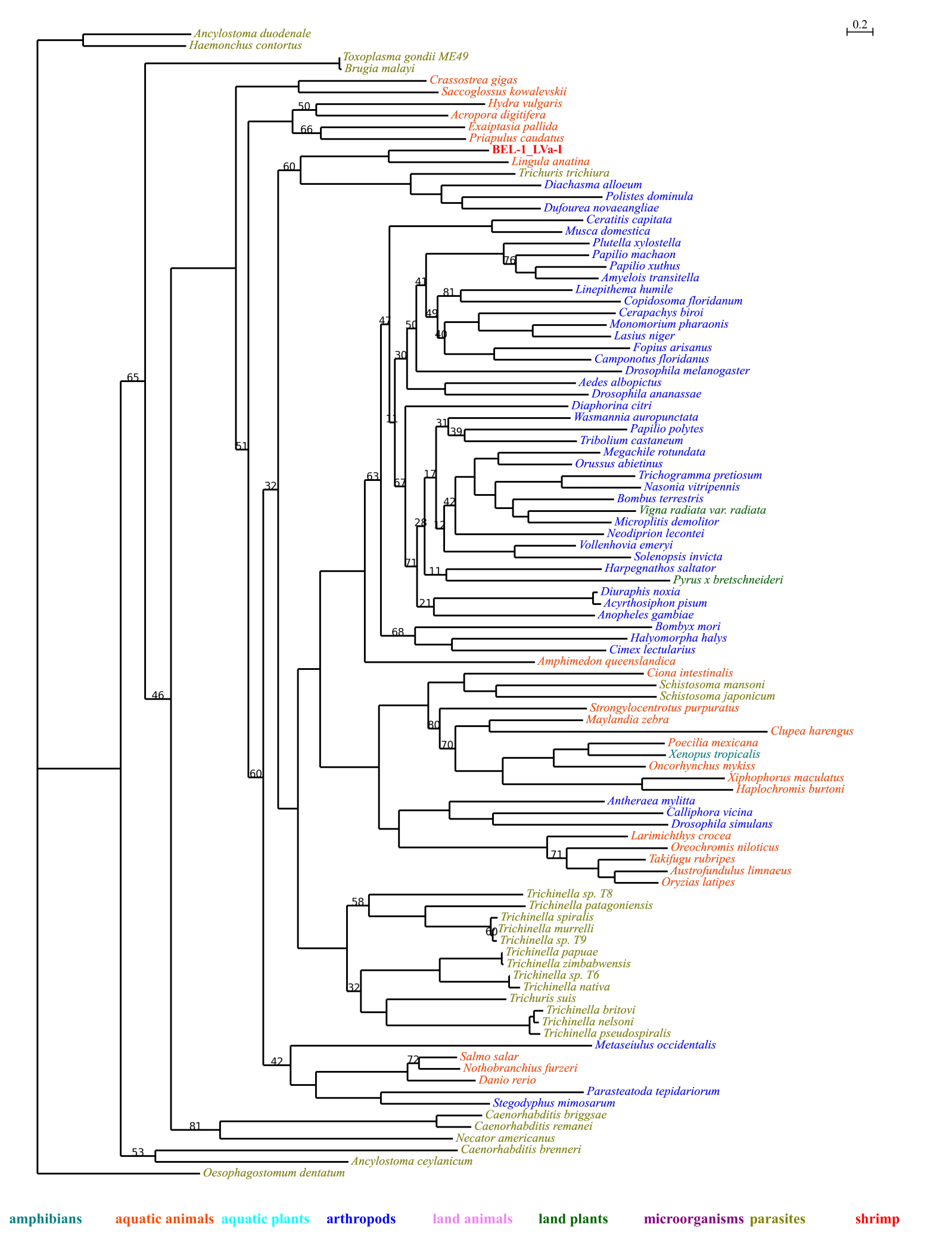


Fig. 1 Phylogenetic tree of BEL-1_LVa-I and its homologues. Only those bootstrap values below 90% were shown (100 replicates); the same below. The *p-value* of approximately unbiased (AU) test is 0.729.


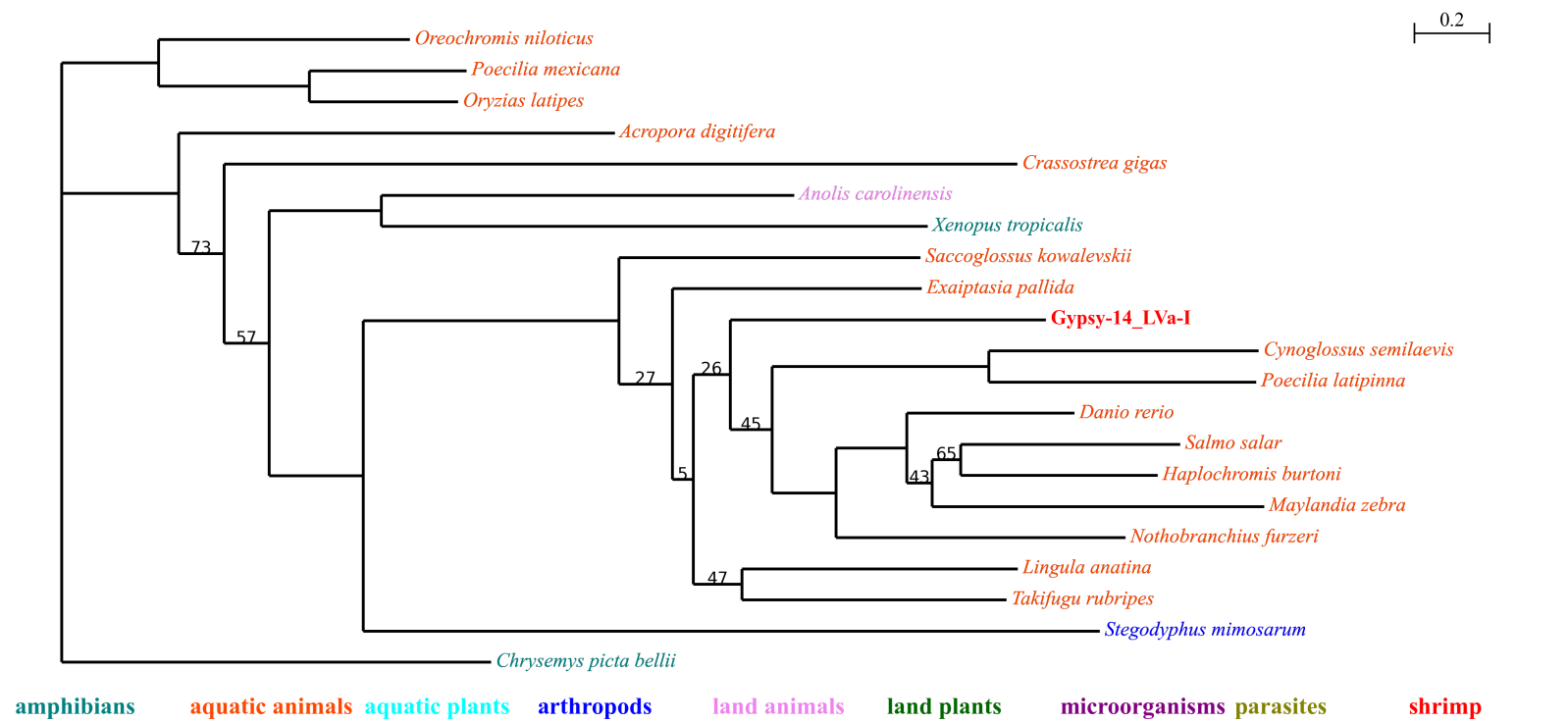


Fig. 2 Phylogenetic tree of Gypsy-14_LVa-I and its homologues. The *p-value* of AU test is 0.770.


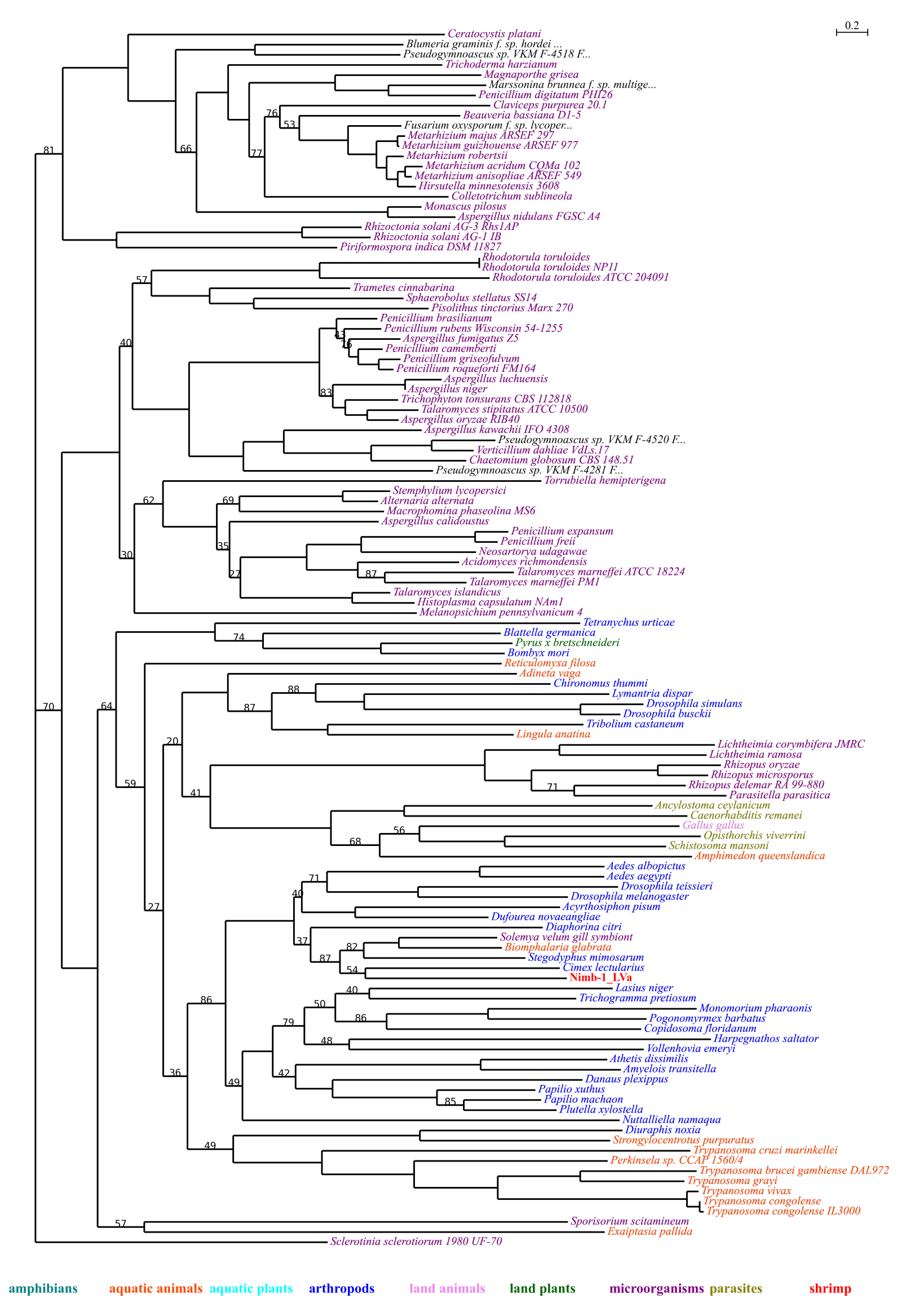


Fig. 3 Phylogenetic tree of Nimb-1_LVa and its homologues. The *p-value* of AU test is 0.760.


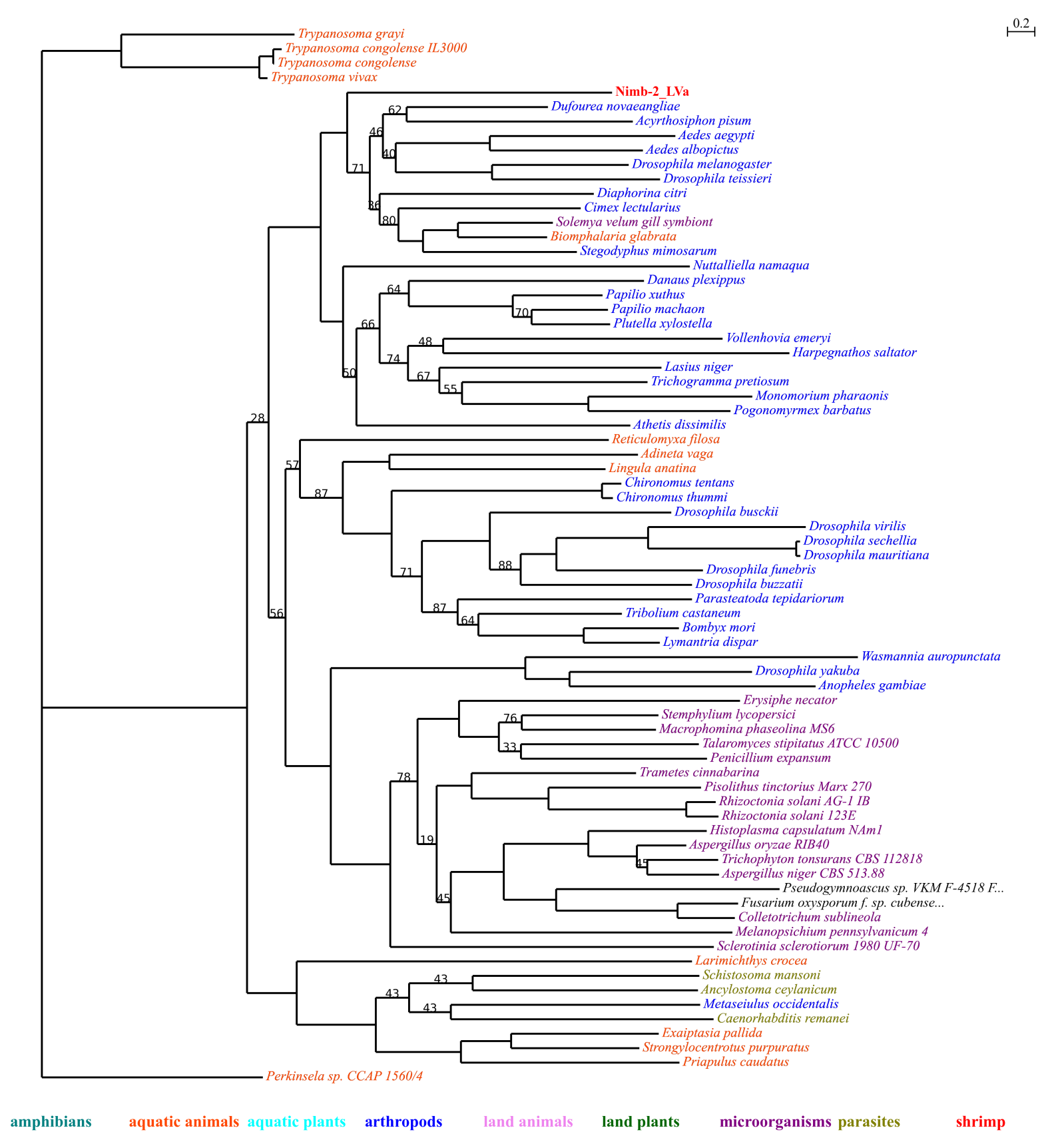


Fig. 4 Phylogenetic tree of Nimb-2_LVa and its homologues. The *p-value* of AU test is 0.899.


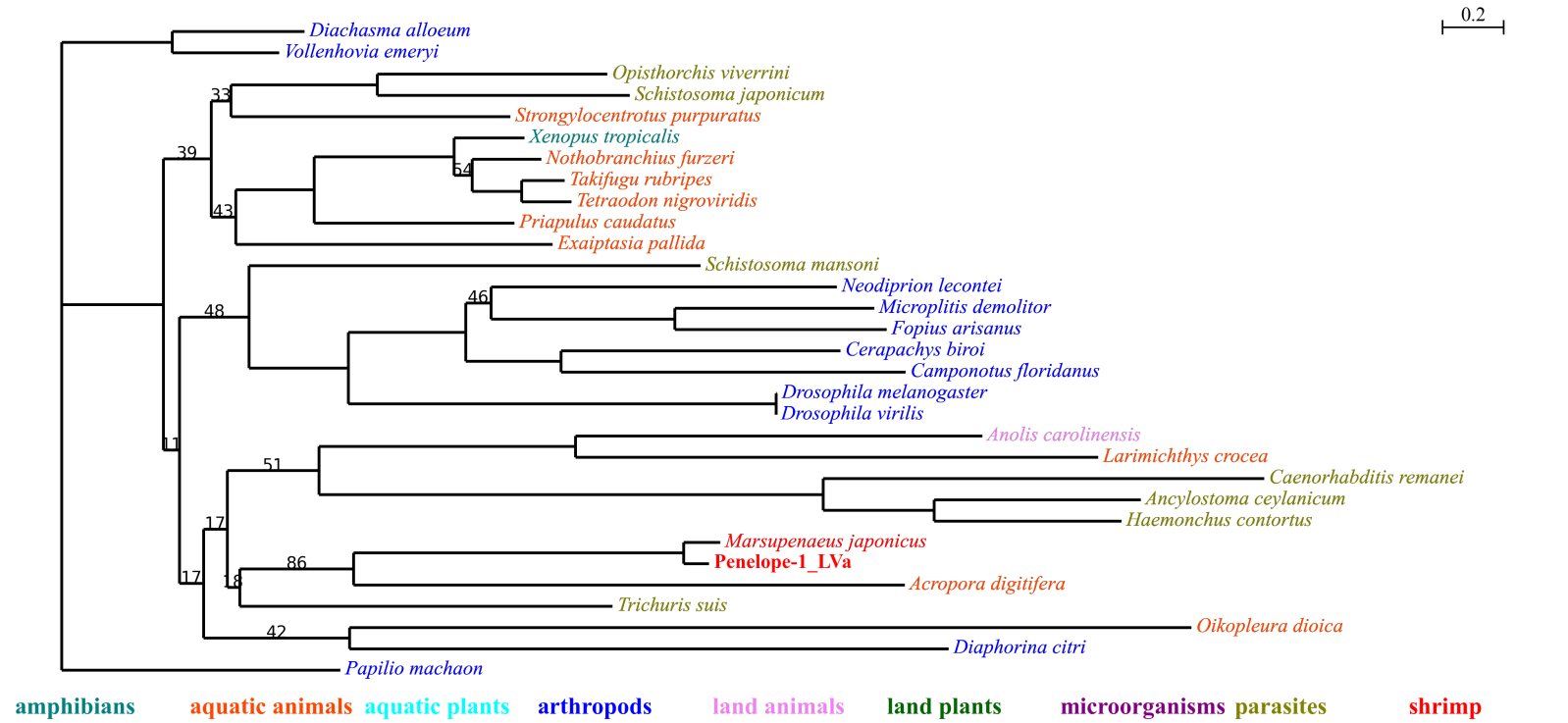


Fig. 5 Phylogenetic tree of Penelope-1_LVa and its homologues. The *p-value* of AU test is 0.926.


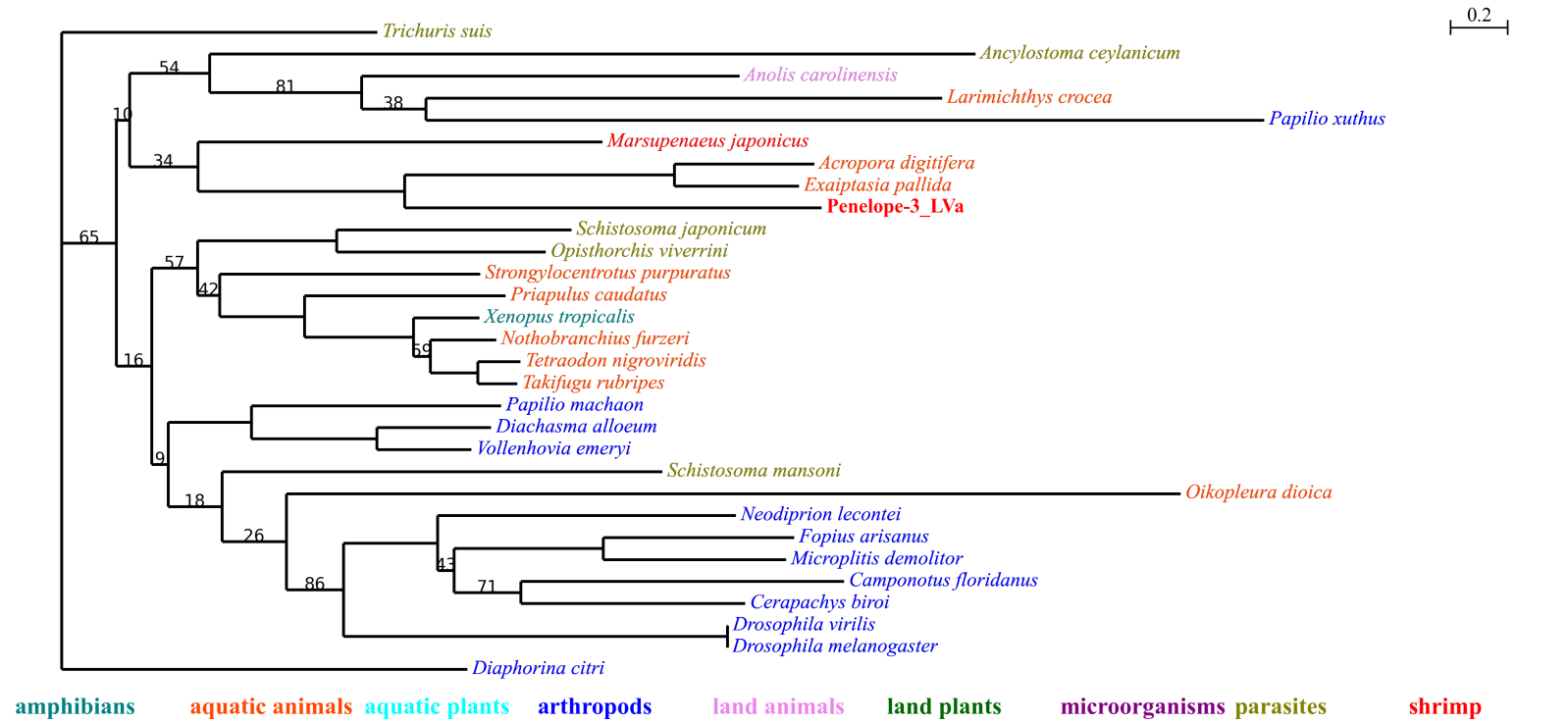


Fig. 6 Phylogenetic tree of Penelope-3_LVa and its homologues. The *p-value* of AU test is 0.875.


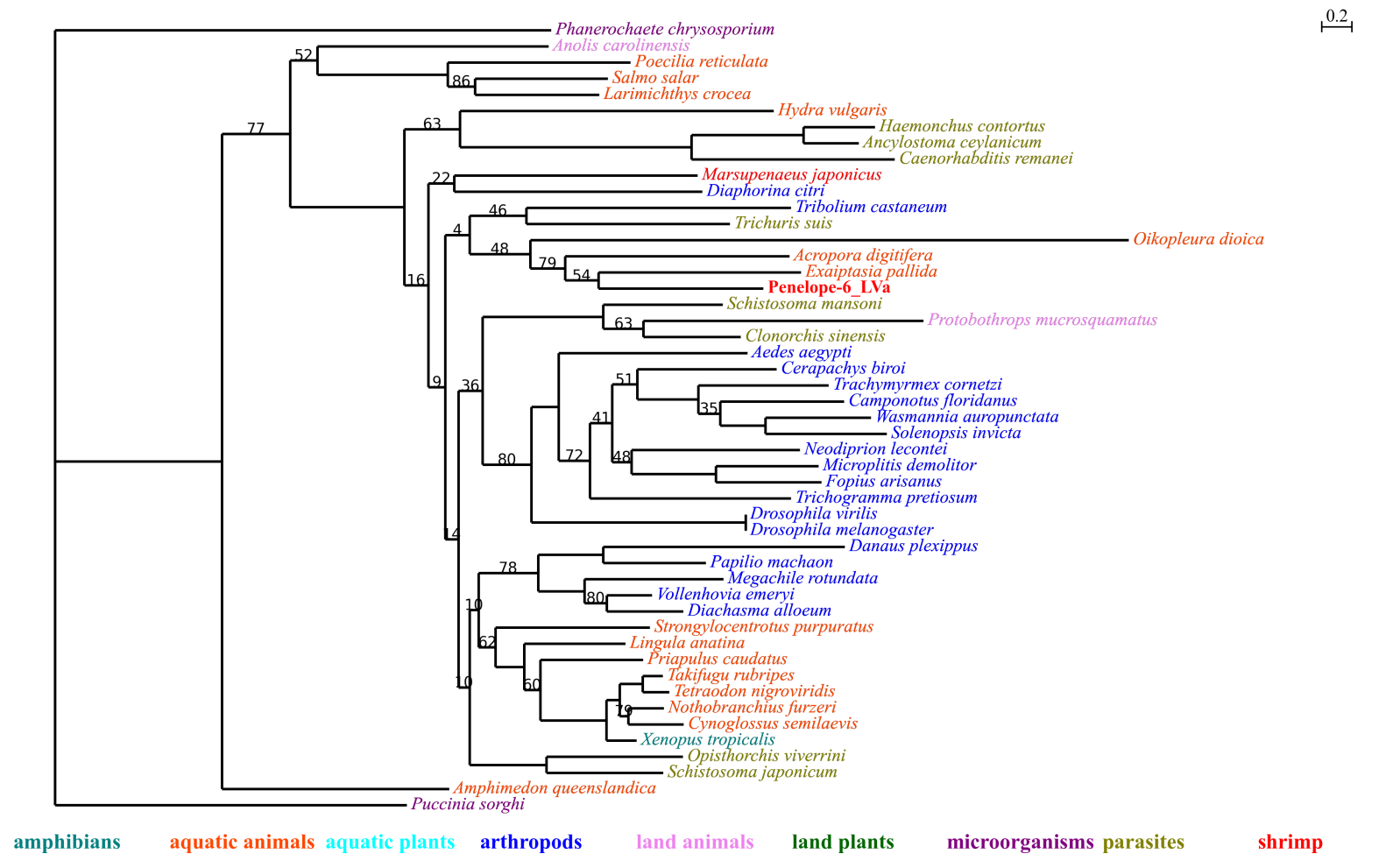


Fig. 7 Phylogenetic tree of Penelope-6_LVa and its homologues. The *p-value* of AU test is 0.812.


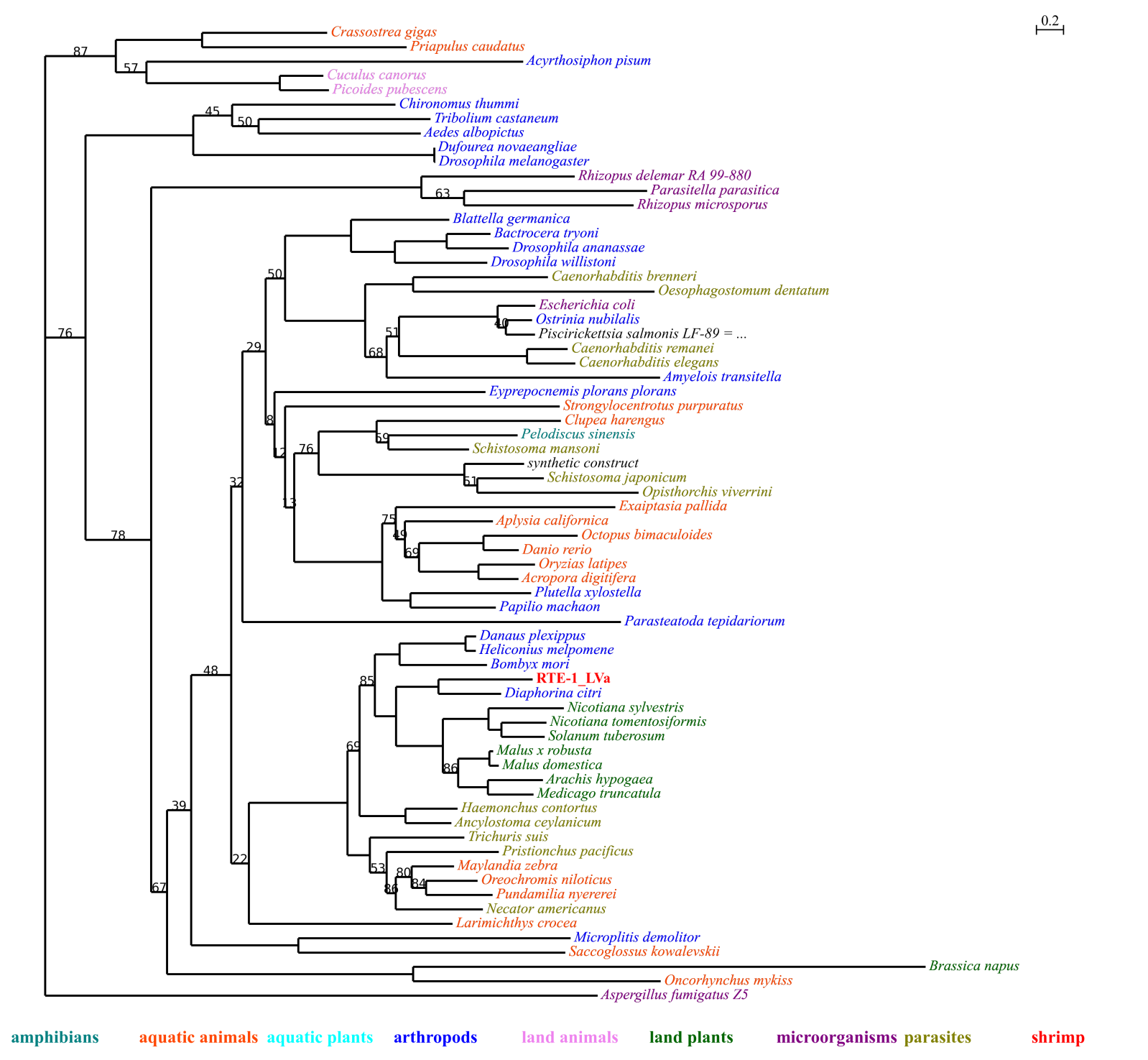


Fig. 8 Phylogenetic tree of RTE-1_LVa and its homologues. The *p-value* of AU test is 0.893.


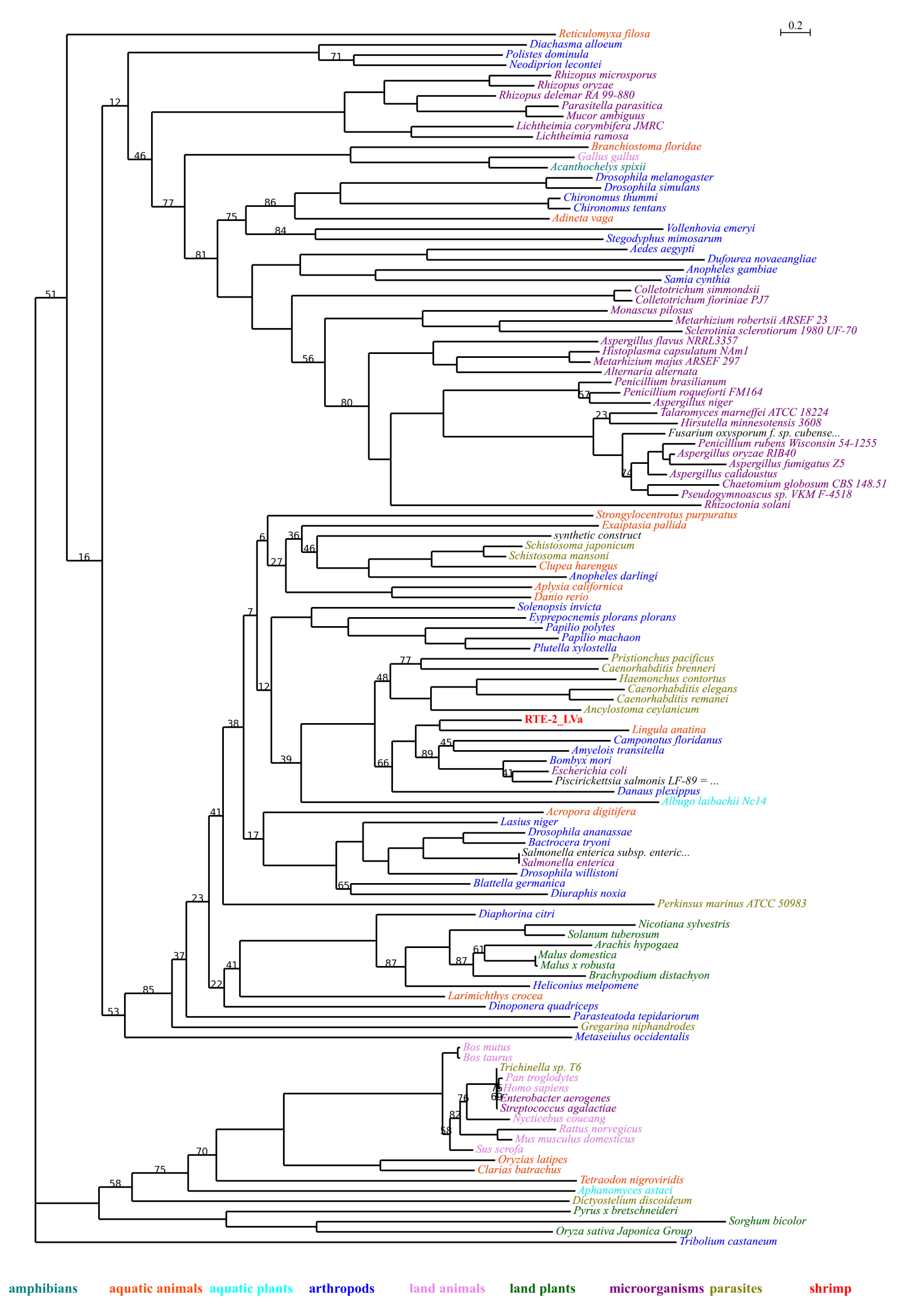


Fig. 9 Phylogenetic tree of RTE-2_LVa and its homologues. The *p-value* of AU test is 0.805.


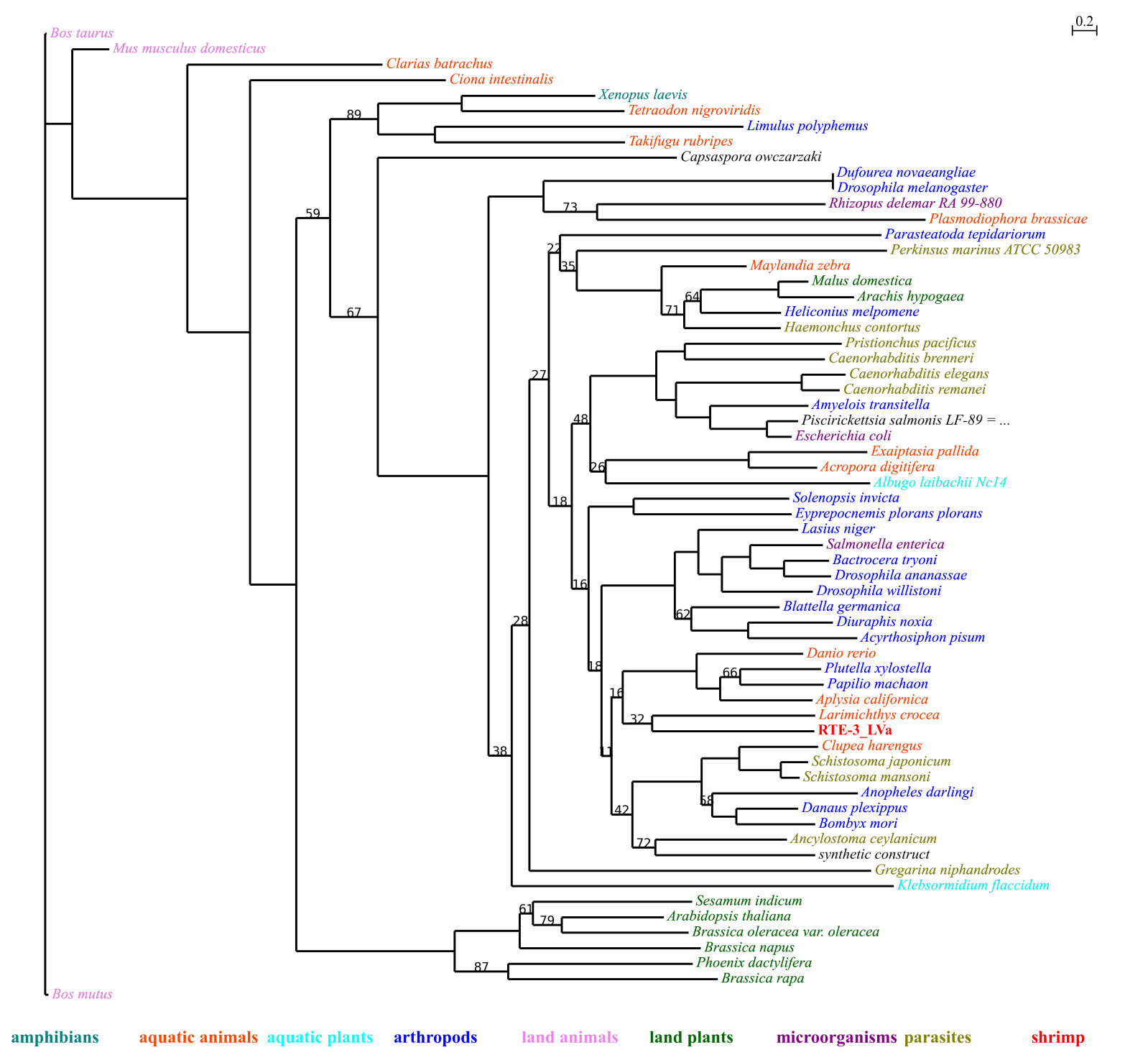


Fig. 10 Phylogenetic tree of RTE-3_LVa and its homologues. The *p-value* of AU test is 0.813.
