# Supplementary figures and images for: Close ecological relationship among species facilitated horizontal transfer of retrotransposons
Source: BMC Evol Biol. 2016 Oct 7;16:201. doi: 10.1186/s12862-016-0767-0 (PMC5055719; doi:10.1186/s12862-016-0767-0)

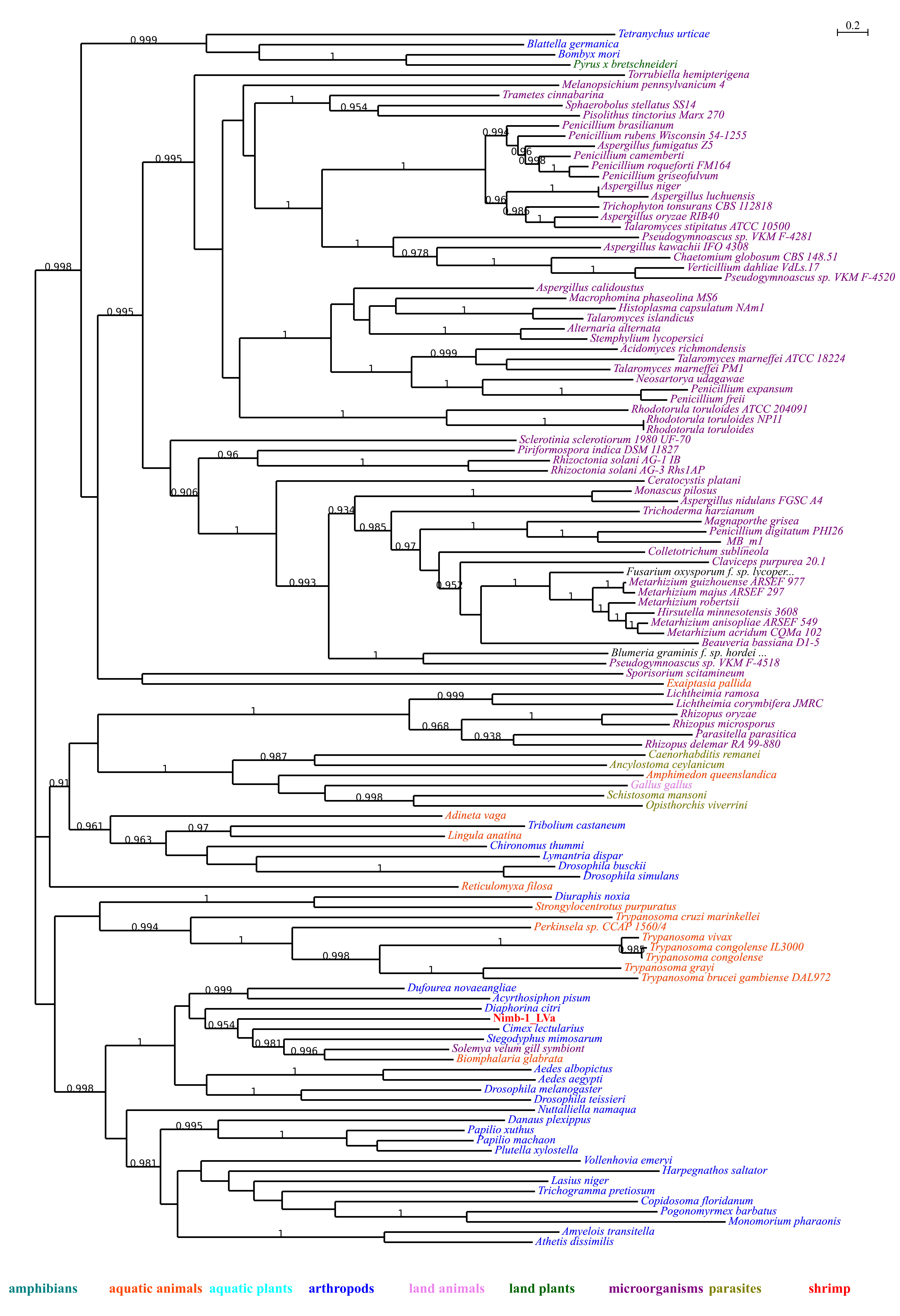

Supplement: Additional file 4: — Phylogenetic tree of Nimb-1_LVa and its homologues. (PNG 2283 kb) [file 12862_2016_767_MOESM4_ESM.png]

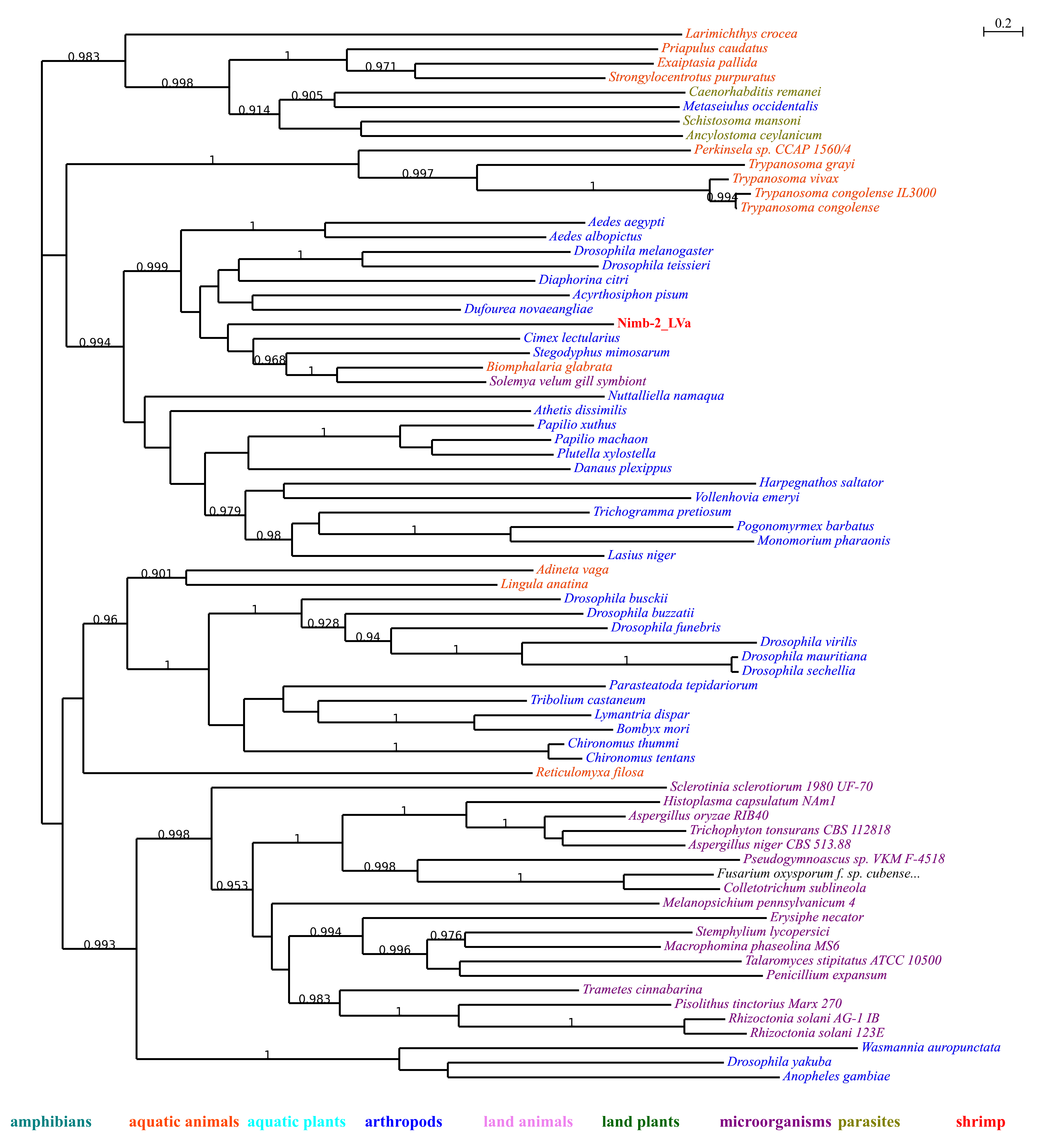

Supplement: Additional file 5: — Phylogenetic tree of Nimb-2_LVa and its homologues. (PNG 1345 kb) [file 12862_2016_767_MOESM5_ESM.png]

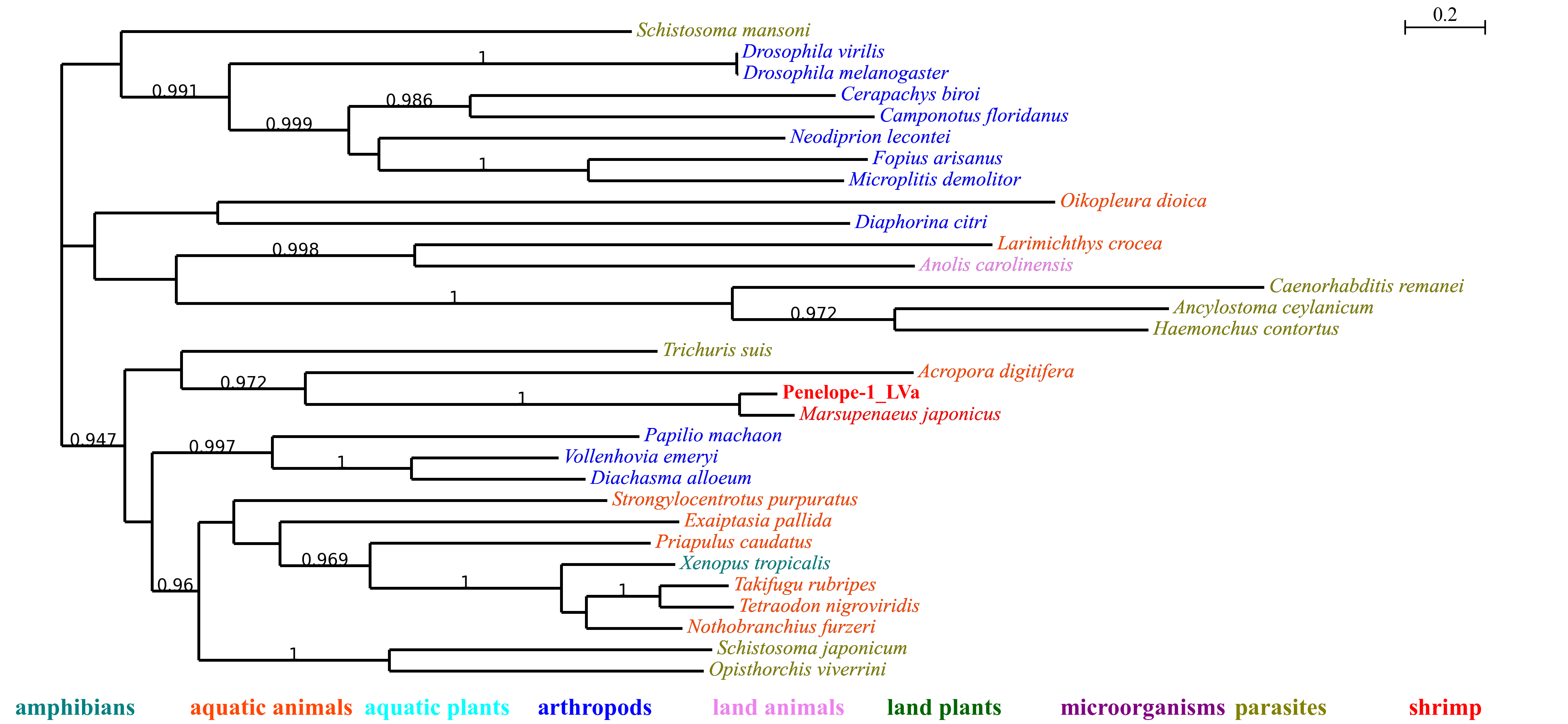

Supplement: Additional file 8: — Phylogenetic tree of Penelope-6_LVa and its homologues. (PNG 854 kb) [file 12862_2016_767_MOESM8_ESM.png]

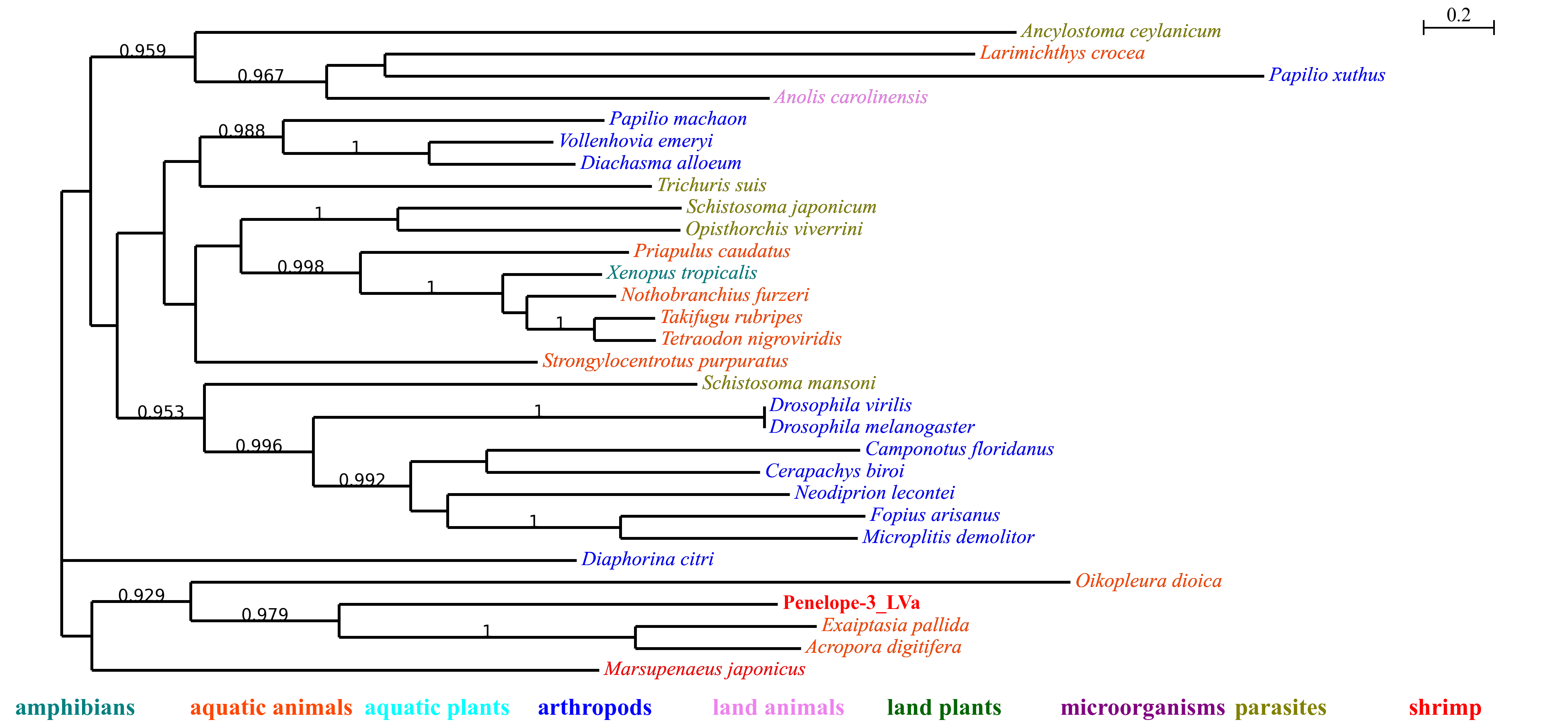

Supplement: Additional file 9: — Phylogenetic tree of RTE-2_LVa and its homologues. (PNG 2080 kb) [file 12862_2016_767_MOESM9_ESM.png]

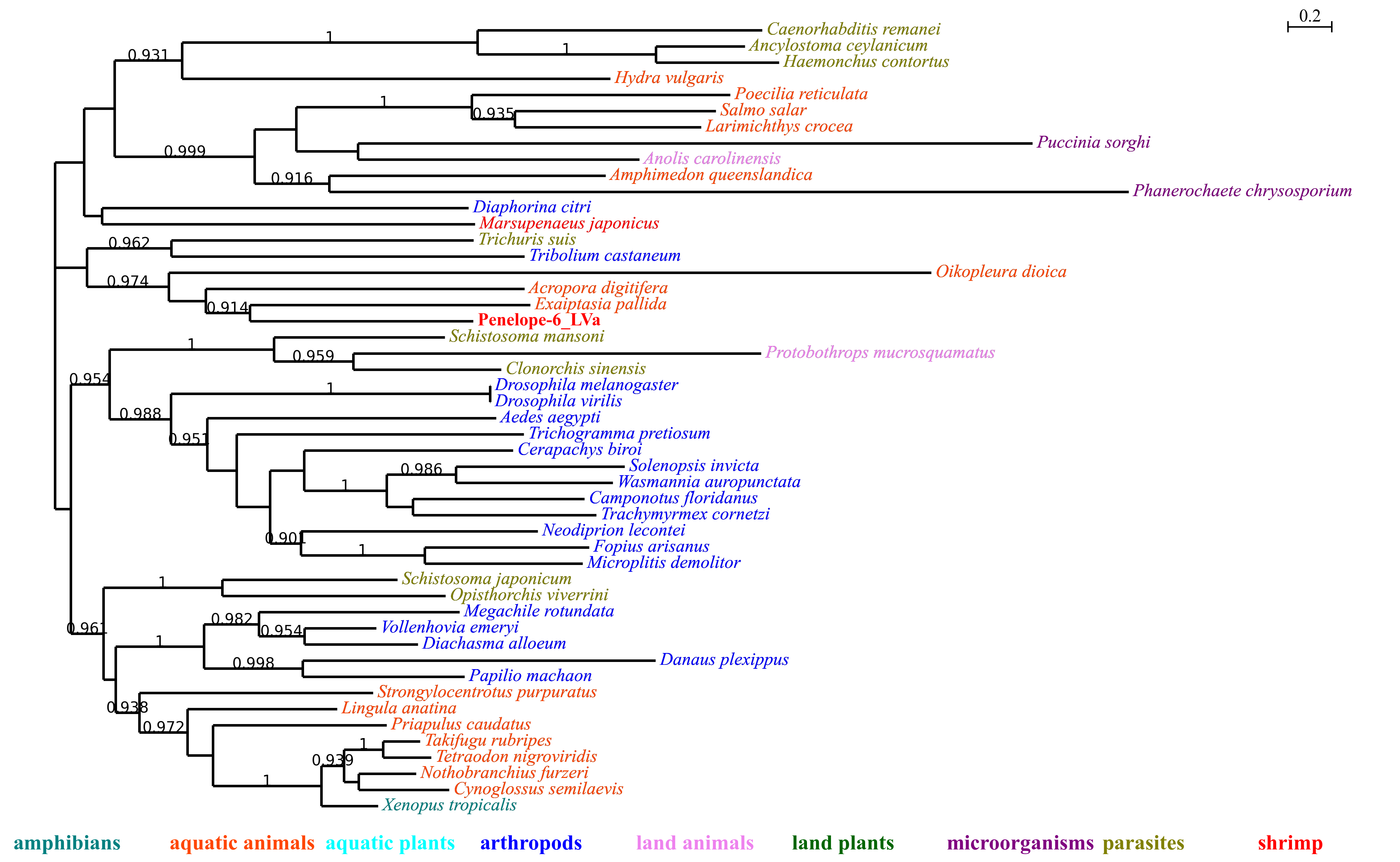

Supplement: Additional file 10: — Phylogenetic tree of RTE-3_LVa and its homologues. (PNG 1074 kb) [file 12862_2016_767_MOESM10_ESM.png]
