# Supplementary material for: Close ecological relationship among species facilitated horizontal transfer of retrotransposons
Source: BMC Evol Biol. 2016 Oct 7;16:201. doi: 10.1186/s12862-016-0767-0 (PMC5055719; doi:10.1186/s12862-016-0767-0)
Supplement: Additional file 11: — Expression change of Rab7 gene in response to VP28 stimulation. (DOCX 16 kb) [file 12862_2016_767_MOESM11_ESM.docx]

The sequence of *L. vannamei* Rab7 gene was downloaded from NCBI (GenBank ID: FJ811529.1). Through local blastn search, we determined that there is only one unique transcript of Rab7 gene, the sequence ID of which is comp10941_c0_seq1. The expression change (read counts) of this sequence in response to VP28 is as follows:

|  | Replicate 1 * norm.factors^a^ | Replicate 2 * norm.factors | Sum |
| --- | --- | --- | --- |
| Blank | 274 * 1.05 | 824 * 1.01 | **1120** |
| Control | 229 *1.15 | 1652 * 1.07 | **2031** |
| Single VP28 | 517 * 1.03 | 1064 * 0.98 | **1575** |
| Successive VP28 | 298 * 0.89 | 629 * 0.84 | **794** |

^a^ norm.factors are obtained from Additional file 1.

Evidently, the expression change of Rab7 gene in control, single VP28 and successive VP28 groups showed the same trend as HTT transcripts in Figure 6C.
